# Sturgeon gut development: a unique yolk utilization strategy among vertebrates

**DOI:** 10.3389/fcell.2024.1358702

**Published:** 2024-05-30

**Authors:** Mujahid Ali Shah, Xuan Xie, Marek Rodina, Jan Stundl, Ingo Braasch, Radek Šindelka, Małgorzata Rzepkowska, Taiju Saito, Martin Pšenička

**Affiliations:** ^1^ Faculty of Fisheries and Protection of Waters, South Bohemian Research Center of Aquaculture and Biodiversity of Hydrocenoses, University of South Bohemia in Ceske Budejovice, Vodnany, Czechia; ^2^ Division of Biology and Biological Engineering, California Institute of Technology, Pasadena, CA, United States; ^3^ Department of Integrative Biology and Ecology, Evolution, and Behavior Program, Michigan State University, East Lansing, MI, United States; ^4^ Laboratory of Gene Expression, Institute of Biotechnology of the Czech Academy of Sciences, Vestec, Czechia; ^5^ Department of Ichthyology and Biotechnology in Aquaculture, Warsaw University of Life Sciences, Warsaw, Poland; ^6^ South Ehime Fisheries Research Centre, Ehime University, Matsuyama, Japan

**Keywords:** sturgeon, gut–endoderm, holoblastic cleavage, meroblastic cleavage, vertebrate evolution

## Abstract

In vertebrates, maternally supplied yolk is typically used in one of two ways: either intracellularly by endodermal cells or extracellularly via the yolk sac. This study delves into the distinctive gut development in sturgeons, which are among the most ancient extant fish groups, contrasting it with that of other vertebrates. Our observations indicate that while sturgeon endodermal cells form the archenteron (i.e., the primitive gut) dorsally, the floor of the archenteron is uniquely composed of extraembryonic yolk cells (YCs). As development progresses, during neurulation, the archenteric cavity inflates, expands laterally, and roofs a semicircle of YCs. By the pharyngula stage, the cavity fully encompasses the YC mass, which begins to be digested at the hatching stage. This suggests a notable deviation in sturgeon gut development from that in other vertebrates, as their digestive tract initiates its function by processing endogenous nutrition even before external feeding begins. Our findings highlight the evolutionary diversity of gut development strategies among vertebrates and provide new insights into the developmental biology of sturgeons.

## 1 Introduction

**FIGURE 1:**
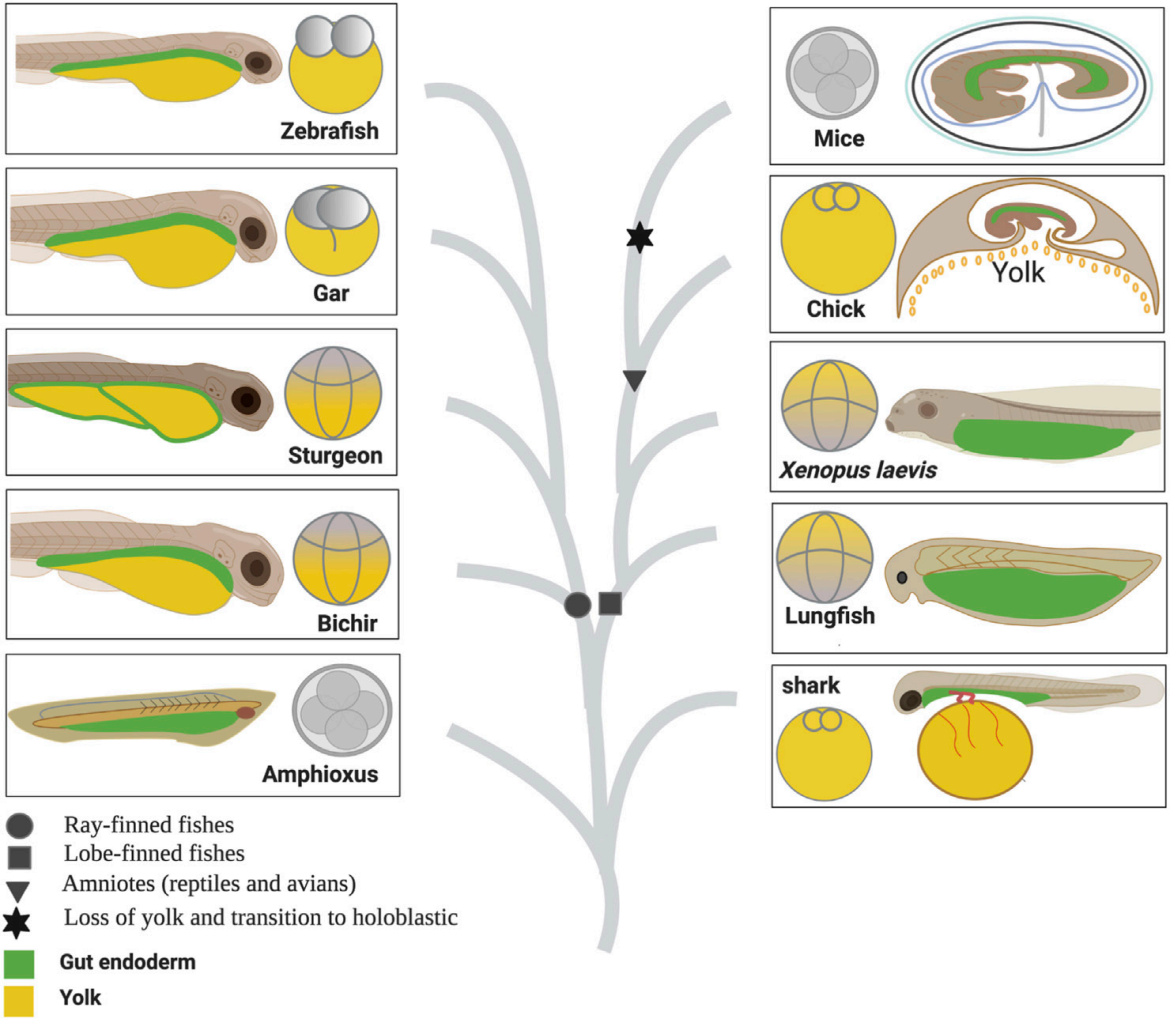
Comparison of egg cleavage patterns and gut anatomy among vertebrates based on their phylogeny relationship. Figure 1 represents the evolution of the egg cleavage pattern and gut development among vertebrates. The illustration has been created based on literature review and current findings (Rosenquist, 1971; Lawson et al., 1986; Lawson and Schoenwolf, 2003; Ober et al., 2003; Tremblay and Zaret, 2005; Kimura et al., 2007; Tam et al., 2007; Takeuchi et al., 2009b; Zorn and Wells, 2009; Kemp, 2011).

In all vertebrates, the three germ layers 1) endoderm, 2) mesoderm, and 3) ectoderm give rise to the entire organism. The nervous system, neural crest derivatives, and skin develop from the ectoderm. The heart, kidney, gonads, gut muscles, and blood-forming tissues develop from the mesoderm. The respiratory and gastrointestinal tract and all of their associated organs develop from the endoderm (Gilbert, 2010). Studies on vertebrate model organisms have extensively described which cells in the embryo give rise to the endoderm and how those cells form a primitive gut tube (Wallace and Pack, 2003; Zorn and Wells, 2009; Gilbert, 2010). However, cross-species comparisons among vertebrates, including fishes (non-teleost and teleost) and tetrapods (amphibians, reptiles including birds, and mammals), are crucial for understanding the gut–endoderm morphogenesis and its evolution (Figure 1).

Living vertebrates are classified into two major groups: jawless (Agnatha) and jawed vertebrates (Gnathostomata, comprising Chondrichthyes and Osteichthyes). Osteichthyes (bony fishes) are further divided into two categories: ray-finned fishes including teleosts and lobe-finned fishes which include tetrapods. In the lobe-finned lineage, holoblastic (complete) cleavage occurs in the eggs of lungfish and amphibians, whereas reptiles including birds undergo meroblastic or incomplete cleavage (Takeuchi et al., 2009b). In ray-finned fishes, teleosts evolved a meroblastic cleavage pattern. On the other hand, non-teleost fishes, including bichir, paddlefish, and sturgeon, retained holoblastic cleavage, whereas the holosteans (gars and bowfin) have intermediate morphologies and represent an obvious transition in cleavage pattern from holoblastic toward meroblastic (for details, see Bolker, 1993; 1994; Long and Ballard, 2001; Takeuchi et al., 2009b; Diedhiou and Bartsch, 2009).

In the lobe-finned lineage, the well-studied holoblastically cleaved embryo of the *Xenopus* frog develops the endoderm from the vegetal blastomeres (Gilbert, 2010). During gastrulation, the embryo of *Xenopus* develops the archenteron or the primitive gut. The entire gut is developed from the archenteron (including dorsal and ventral parts) and underlaid with mesenchymal cells. The maternally supplied yolk is stored and later digested intracellularly in the endodermal cells in the form of yolk platelets (Keller, 1981; Shih and Keller, 1994; Zorn and Wells, 2009). Comparatively, due to increased egg size and yolk-rich vegetal blastomeres, the ventral part of the archenteron in the embryos of the directly developing frog *Eleutherodactylus coqui* is made up of nutritional yolk cells (YCs), which do not contribute to gut development. The increased amount of yolk in frogs is reminiscent of the situation in reptiles and birds (Buchholz et al., 2007; Takeuchi et al., 2009b).

Following the increased yolk mass, reptiles and birds evolved a meroblastic cleavage pattern, resulting in a flattened blastodisc on the yolk mass and displaying a gastrulation pattern that differs significantly from that in amphibian embryos (Zorn and Wells, 2009). Placental mammals, on the other hand, lose the yolk, which leads to a reversal of the transition of cleavage patterns from meroblastic to holoblastic, and develop in the uterus, while retaining the same gastrulation pattern conserved in other amniotes (Takeuchi et al., 2009b). During gastrulation in chicken and mice, the epiblast (primitive ectoderm) undergoes an epithelial-to-mesenchymal transition and migrates through the primitive streak, which is analogous to the dorsal blastopore lip in amphibians, and gets incorporated in the mesoderm (middle) or endoderm (outer) layer (Zorn and Wells, 2009). The cells of the presumptive definitive endoderm invade and displace an outer layer of extraembryonic tissue cells, the hypoblast in chicken and the visceral endoderm in mouse, which form supporting structures such as the yolk sac. Lineage tracing studies of the endoderm have described how a two-dimensional sheet of cells forms the primitive gut tube (Rosenquist, 1971; Lawson et al., 1986; Lawson and Schoenwolf, 2003; Tremblay and Zaret, 2005; Kimura et al., 2007; Tam et al., 2007).

In teleost fishes, similarly to reptiles/birds, eggs divide partially. The germ ring is formed during gastrulation by the thickening deep cells at the leading edge of the vegetally expanding blastoderm. The germ ring’s deep cells undergo involution to form two layers: the epiblast and the hypoblast. The epiblast gives rise to ectodermal cell lines, while the hypoblast contributes to the formation of the embryonic endoderm (Ober et al., 2003; Gilbert, 2010). The entire gut develops and lies on the yolk sac; the yolk is substantially used up, and feeding commences, allowing for nutritional intake to be processed in the gut (Kimmel et al., 1995).

In contrast, holoblastically cleaved embryos of non-teleost fishes such as sturgeon and bichir share many developmental similarities with *Xenopus.* It has been reported that bichir embryos develop an archenteron that is very similar to that of *Xenopus*, yet the ventral part of the archenteron is made up of extra-embryonic YCs (Bolker, 1993; 1994; Collazo et al., 1994; Takeuchi et al., 2009a). The entire gut of bichirs is established as a tubular structure with mesenchymal cells underneath, and a YC mass exists on the ventral side of the embryo. The YCs are not underlaid with the mesenchymal cells, as in *Xenopus*, but it is directly surrounded by the surface ectoderm as in the yolk sac in teleosts (zebrafish) and is, therefore, extra-embryonic. A similar developmental pattern has also been observed in the frog *E. coqui* (*E. coqui*) (Wallace and Pack, 2003; Buchholz et al., 2007; Takeuchi et al., 2009b).

Recently, we reported that the YCs of sturgeon embryos are extraembryonic and serve only to provide nutrition (Shah et al., 2022), such as the yolk of teleosts (zebrafish) and the YCs of bichir, agnathan lampreys (Petromyzontidae)—an extant lineage of jawless fishes, and the YCs of *E. coqui* (Buchholz et al., 2007; Takeuchi et al., 2009b). Moreover, reports indicate that during the post-hatching stage, the alimentary canal of sturgeon larvae becomes filled with yolk reserves. However, a detailed description of how sturgeons develop their gut to utilize these yolk reserves remains poorly understood (Ballard and Needham, 1964; Ballard and Ginsburg, 1980; Buddington and Doroshov, 1986). Hence, the current study aims to comprehensively elucidate the intricate developmental trajectory of the sturgeon gut. To achieve this, a multifaceted approach including resin histology, high-resolution electron microscopy, molecular analysis involving the expression profile of the conserved marker gene *sox17*, and sophisticated fate mapping through cell labeling with the carboxy-CDCFDA dye was employed. Furthermore, we compared sturgeon gut development to that of other vertebrate taxa, including those from lobe-finned (*Xenopus*, chicken, and mice) and ray-finned (bichir, gar, and zebrafish) lineages, to better understand its context in gnathostome evolution (Figure 1).

## 2 Materials and methods

### 2.1 Samples

The specimens of bichir, sterlet, gar, zebrafish*, Xenopus,* chick, and mouse were prepared as follows: zebrafish and sterlet sturgeon were bred at Genetic Fisheries Centre, Faculty of Fisheries and Protection of Waters in Vodnany, and the stages were selected according to Dettlaff (1993) and Kimmel et al. (1995). Gar specimens were raised and prepared at the Department of Integrative Biology, College of Natural Science, Michigan State University, as described previously (Braasch et al., 2014), and the stages were selected according to Long and Ballard (2001). Bichir specimens were prepared at the Department of Zoology, Charles University, and the stages were selected according to Diedhiou and Bartsch (2009). *Xenopus* specimens were prepared at the Laboratory of Gene Expression, and the stages were selected according to https://www.xenbase.org/entry/. Mouse and chicken specimens were prepared at the Department of Animal Sciences, Warsaw University of Life Science, and the developmental stages were selected according to Hamburger and Hamilton (1951) and Kaufman MH (1992).

### 2.2 Histology and transmission electron microscopy

To study the morphological development of gut formation in sturgeon and other taxa, plastic sections were prepared using histology to retain the structure of the tissues with lipids intact. Specimens in triplicates from all animals were fixed in Bouin’s fixative or 4% PFA for 24 h and then stored in 70% EtOH and dehydrated in a series of alcohol (75%, 75%, 90%, and 100%). Then, they were embedded in JB-4 resin, sectioned dorsoventrally at 5 μm, stained with H&E, mounted with DPX, and observed under the light microscope. The images were captured using an Olympus microscope. The histology sections of bichirs were obtained from the Department of Zoology, Charles University, Prague, Czech Republic (for details, see Stundl et al., 2020).

In addition, electron microscopy was used to examine the ultrastructure of the germ layers and YCs in sturgeon. PFA-fixed specimens at stages 24, 32, 36, and 40 were rinsed three times with PBS before being fixed for 2 hours with osmium tetroxide. Samples were dehydrated using an acetone series and embedded in Spurr’s resin (TAAB Laboratories Equipment Ltd.). Samples were sectioned dorsoventrally on a Porter–Blum MT-1 ultramicrotome (DuPont Sorval) with a diamond knife and mounted on formvar-coated slot grids. Sections were stained with uranyl acetate and lead citrate and examined using a JEOL 1011 electron microscope (JEOL) (Saito et al., 2014).

### 2.3 Immunohistochemistry of FITC-labeled embryos of sterlet sturgeon

We injected 10% FITC dextran 500000 MW (FD5) into the vegetal pole at the developing stage 10 (blastula stage) and speculated that vegetal blastomeres contain extraembryonic YCs, which will be encompassed by the gut (Shah et al., 2022). The FD5-labeled embryos in triplicates were allowed to develop until stage 38 and were then fixed overnight in Bouin’s fixative and embedded in paraffin. Serial sections of 8 µm were cut and placed on glass microscope slides with 0.01% poly-L-lysine. FITC labeling was detected using an anti-FITC antibody (Invitrogen, #71–1900) and visualized with diaminobenzidine by the ABC reaction (Vector), as described in the manufacturer’s protocol. The sections were counterstained with hematoxylin and eosin. Pictures were taken using an Olympus microscope. For fibronectin labeling, samples from stages 20–22, 26–28, and 30–32 were prepared similarly to the method used for ISH-HCR. The protocol was followed with minor modifications, as described by Shah et al., 2022. Antigen retrieval was performed using 1X citrate buffer, followed by a 4-h block in an antibody dilution buffer (Dako S0809). The samples were then incubated overnight at 4°C with fibronectin antibody (Dako QO149) at a 1:500 dilution and, subsequently, with the secondary anti-rabbit immunoglobulin G–fluorescein isothiocyanate (FITC; F0382, Sigma) antibodies. Sections were then covered with Fluoroshield™ containing DAPI (Sigma ab104139). Imaging was conducted using a fluorescence microscope (Olympus SZ-12).

#### 2.3.1 *In situ* hybridization chain reaction (HCR)

Multiplexed, quantitative, high-resolution RNA fluorescence *in situ* hybridization (HCR-FISH) was used as instructed by Molecular Instrument (MI), *imaging and molecules of life*™. A *Sox17* probe was created as Ar-LOC117397484 targeting 176–1,357 bp of *Acipenser ruthenus* transcription factor *Sox-17-alpha-A* (GeneID: 117394216). A *β-actin* probe, named actb1, targeting 151–1,278 bp of *A. ruthenus* beta actin-1 (Gene ID: 117431529) was used as a positive control. We used *in situ* staining on paraffin-embedded section. The protocol was followed as instructed by MI for zebrafish FFPE-samples. Embryos at the required developmental stages were fixed in 4% PFA overnight at 4°C, dehydrated in an ethanol series followed by xylene, embedded in paraffin, sectioned at 5 μm, baked at 60°C for 1 hour, and deparaffinized in xylene and 100% ethanol. Specimens were then rehydrated with an ethanol series, followed by antigen retrieval according to the HCR-FISH protocol. After treatment with protease-K 1 μL.mL^-1^ for 10 min at 38°C in a humidified chamber, hybridization with target probes and amplification was performed. The samples were mounted with Fluoroshield 4′,6-diamidino-2-phenylindole (DAPI), covered with a coverslip, observed under a confocal microscope Olympus FV 3000, and processed with cellSens Olympus software.

### 2.4 Lineage tracing of mesendodermal cells

To label the endodermal cells of sterlet sturgeon and gar embryos, 50 mM of CDCFDA [5-(and-6)-carboxy-2′,7′-dichlorofluorescein diacetate, cat. no.: 22026, AAT BioQuest, Inc.] stock was prepared by dissolving in DMSO (dimethyl sulfoxide) and stored at −20°C. A working concentration (50–100 μM) was prepared by diluting the stock in a 10% sucrose solution. During the cleavage phase, embryos were manually decapsulated with a forceps and allowed to develop until the early neurula stage (stage 19–20), after which they were placed in an agar-coated Petri dish with a 2-mm hole to position the embryos. The dye was precisely injected into the endoderm of the archenteron through the opened cranial neural tube. The dye passed freely into the adjacent endodermal lining, forming cell membrane-impermeant products that cannot stain the ectoderm situated across the basal lamina; however, in some embryos, the mesoderm was labeled, and those were excluded based on sections (see Supplementary Figure S1). Embryos were analyzed 30 min post-injection and at developmental stages 22–26 under a florescent microscope to ensure the positive injection. Embryos were allowed to develop under dark condition until the gut tube was fully developed. The labeled specimens at stage 38 were anaesthetized in MS222. They were subsequently fixed in 4% PFA under dark conditions and embedded in Tissue-Tek O.C.T and stored at −80°C. For cryosection, the embedded blocks were kept in a cryostat chamber (−17 to −18°C) for 20 min to equilibrate the temperature.

In order to further verify whether the yolk material (YCs) is encompassed by the germ layers, the embryos were immersed in 100 μM concentration of CDCFDA in dechlorinated water in triplicate during stages 16–24, 22–28, and 26–30 (see Figure 4). Subsequently, the embryos were fixed in 4% PFA and sectioned dorsoventrally using JB-4 resin. For the detailed protocol, please refer to Sullivan-Brown et al. (2011).

For both cyro- and resin sections, the thickness of the cut was 8–10 μm at transverse sections. The slices were collected on poly-L-lysine-coated slides and stained with Fluoroshield™ with DAPI (Sigma) and covered with the cover slip. Images were taken using a fluorescence microscope (Olympus SZ-12). Only endoderm-labeled embryos were counted based on the sections (Supplementary Figure S1). For gar endoderm analyses, pictures of the labeled specimens were provided by the Department of Zoology, Charles University, Prague, Czech Republic, and the protocol is described by Minarik et al. (2017).

## 3 Results

### 3.1 Development of the sturgeon archenteron

**FIGURE 2:**
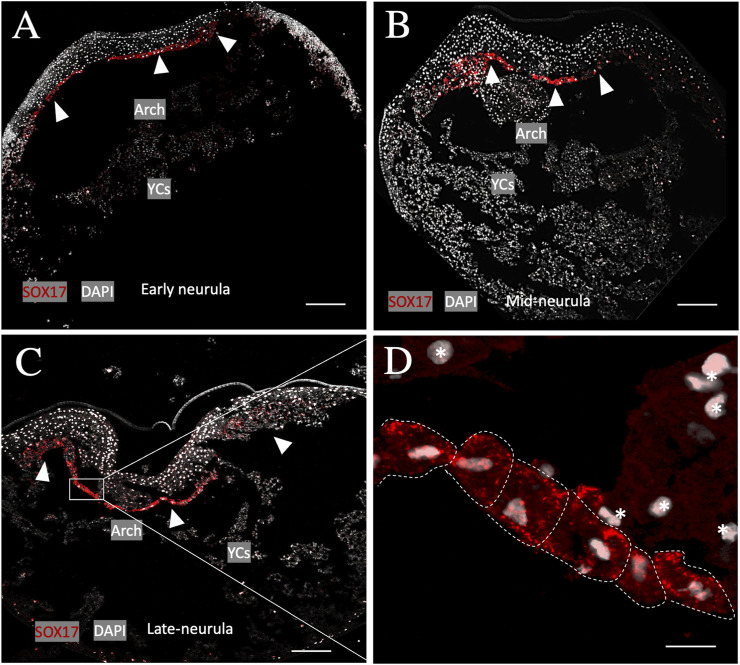
Primitive gut of sturgeon. Staining of the endoderm using marker gene *sox17* from stages 20–24. **(A)** Early neurula (stage 20) shows the archenteron that encompasses the semicircle area of yolk cells. **(B–D)** During mid and late neurula (stage 22–24), a tubular gut on the dorsal position of yolk was not observed. **(D)**. Magnified view of endoderm cells from the archenteron. Arrows indicate the positive signals from endodermal cells of the archenteron, and white dashed lines indicate the endodermal cells with high magnification (insert, stage 24). Red color: sox17, gray color: DAPI, YCs: yolk cells, and star: nuclei from the yolk cells. Arch: archenteron. Scale bars indicate 100 μm and 25 μm in the magnified picture.

**FIGURE 3:**
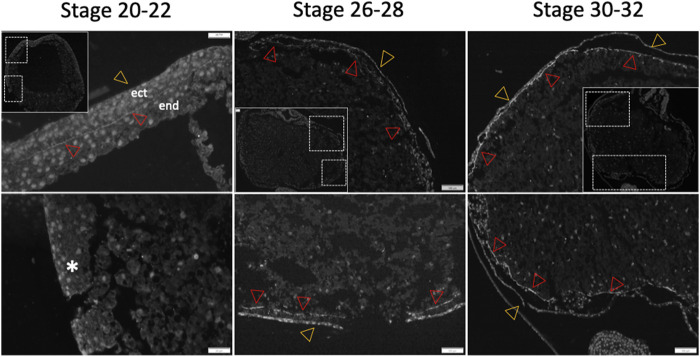
Differential fibronectin staining patterns in sturgeon embryo. Fibronectin staining is used to identify cell and tissue borders during the gut development of sturgeon embryos. During the neurula stage (stages 20–22), fibronectin highlights the borders between cells and tissues, delineating the ectoderm and endoderm on the dorsal side of the embryos. Notably, no staining is observed on the ventral side (see inset). From the pharyngula stage (stages 26–28 onset) through to the pre-hatched larvae stage, the staining continues to mark the borders of cells and tissues (inset for stages 30–32). ect: ectoderm, end: endoderm, star: no staining, red arrow: endodermal border, and yellow arrow: ectodermal border.

To determine whether the archenteron (primitive gut) of sturgeon is formed of dorsoventral endodermal cells (as in *Xenopus*) or if the ventral part consists of extraembryonic YCs (as in bichir), we conducted an *in situ* hybridization experiment using a putative endoderm marker (*sox17*) (Hudson et al., 1997; Takeuchi et al., 2009b; Minarik et al., 2017). The expression analysis of the endodermal gene *sox17* during the early-to-late neurula stage revealed that cells of endodermal origin form the archenteron on the dorsal side of YCs (Figure 2A). The archenteric cavity was inflated and expanded laterally and roofed the semicircle of extraembryonic YCs instead of forming a tubular gut on the top of YCs (Figures 2A–C). The expression of the sox17 marker gene in endodermal cells was detected only from the early neurula to the late neurula stage, and there was no expression in cells from the ventral part, i.e., YCs. In addition, until stage 24 (late neurula), we did not find sox17 expression in endodermal cells on the lateral and ventral side of embryos (Figure 2D). To determine whether endodermal cells were present on the lateral and ventral side during neurulation or had migrated during the pharyngula stage, we employed another staining method using a fibronectin antibody. Fibronectin plays a major role in cell adhesion, growth, migration, and differentiation, and it is crucial for processes such as separating and supporting the organs and tissues of an organism (Minarik et al., 2017). During neurula stages 22–24, fibronectin labeling was found only on the border of endoderm cells, but not on the ventral side (stages 20–22, Figure 3). However, during the pharyngula (stages 26–28) and in the tail-bud (stages 30–32), we observed clear fibronectin labeling on the gut–endoderm border, revealing that endodermal cells encased the yolk during the pharyngula stage (Figure 3).

#### 3.1.1 Gut tube formation: yolk cells (yolk) encompassment by germ layers

**FIGURE 4:**
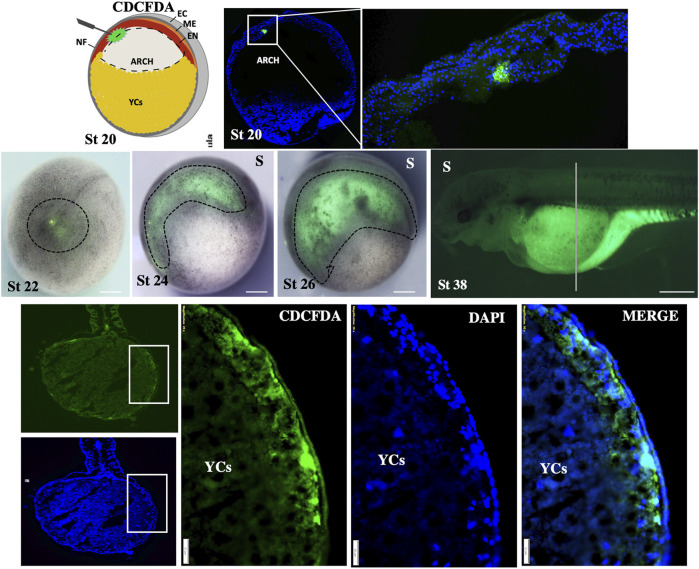
Fate-mapping of gut-endoderm. The CDCFDA dye was precisely injected into the endoderm of archenteron through the opening of the neural tube during early neurula (stage 19–20). After 30 min, embryos were fixed and median-sectioned to ensure positive labeling of endodermal cells (green color shows the dye within the endodermal cells; zoom-out of the rectangular box at stage 20). The embryos show positive labeling at stages 22–26, indicated by dotted lines. The specimens at stage 38 and dorsoventral section of the same stage (38*) show the positive labeling of endoderm cells in green color. Sectioned specimens were counter-stained with DAPI (marked color). Marge image distinguishes the endoderm and ectoderm. D: dorsal view, S: side view, NF: neural plate, EC: ectoderm, ME: mesoderm, and EN: endoderm. Scale bars indicating the 1 mm in stage 38, 200 μm in (transverse section view of stage 38), and 50 μm in (magnified view from the rectangular box of transverse sections).

To investigate the formation of the gut tube, endoderm cells were labeled during early neurula (stage 19–20). To this end, we injected the CDCFDA-dye (fluorescein diacetate) next to the midline of the neural plate to prevent staining of the mesoderm (Figure 4). However, it proved to be quite challenging to prevent mesoderm labeling during endoderm labeling, and we were only able to successfully label a few positive embryos (approximately 12 embryos out of the 120 that were injected). The distinction between the endoderm and mesoderm was made based on the sections (see Supplementary Figure S1). Labeled embryos at the neurula stage showed that the archenteron is located over the roof of YCs (stage 22–24, Figure 4). During the pharyngula stage, labeled cells were observed on the lateral position of developing embryos (stage 26, Figure 4), which suggests that endodermal cells move in a lateroventral direction and develop the gut over the YC region (stage 38, Figure 4).

**FIGURE 5:**
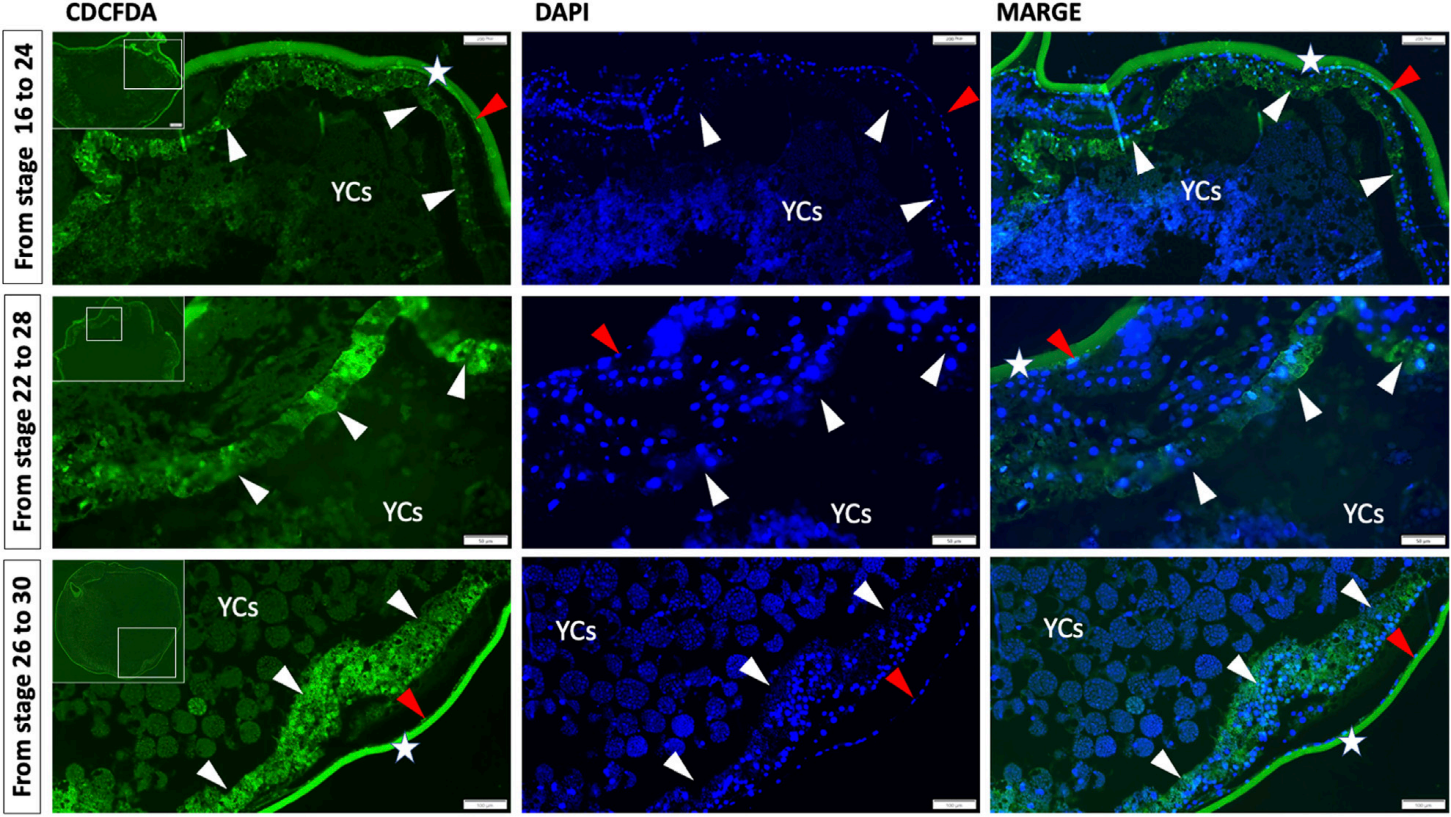
Germ layer (endoderm) development and encompassment of yolk embryos ranging from gastrula to neurula stages (16–26) and pharyngula stages (22–28 and 26–30) were subjected to a pulse and chase experiment using carboxy-CDCFDA. The transverse/anterodorsal sections show that during these stages, endoderm cells developed over the YCs instead of forming a tubular gut on the dorsal side of the yolk. The white arrow indicates the gut endoderm enclosing the YCs, the red arrow indicates the ectoderm, the green color represents CDCFDA labeling, and the blue color represents DAPI. Stars: second chorion with background and YCs: extraembryonic yolk cells. Scale bar, 100 μm = stage 16–24 and 26–30, and 50 μm = stages 22–28.

In addition, to prove our hypothesis that before hatching, the gut (endoderm cells) will enclose the yolk inside, we employed the pulse-chase experiment by immersing the embryos in CDCFDA at a specific time during the development—from the gastrula to neurula stages (stages 16–24, Figure 5) and during the pharyngula stages (stages 22–28 and 26–30, Figure 5). The CDCFDA (fluorescein diacetate) and its derivatives are non-fluorescent molecules that diffuse into cells and are hydrolyzed by intracellular nonspecific esterase to produce fluorescent products (Breeuwer et al., 1995). During these stages, endodermal cells proliferate, and the fluorescent products accumulate only in cells with intact cell membranes. In contrast, YCs after the late blastula stage should be inactive cells (Shah et al., 2022) with leaky membranes and would not be stained. Our pulse-chase experiment revealed that during neurula stages, endodermal cells do not develop around the gut (stages 16–24, Figure 5); however, the endodermal cells continue to proliferate ventrally to form a gut around the YCs during pharyngula (stages 22–28 and 26–30, Figure 5).

**FIGURE 6:**
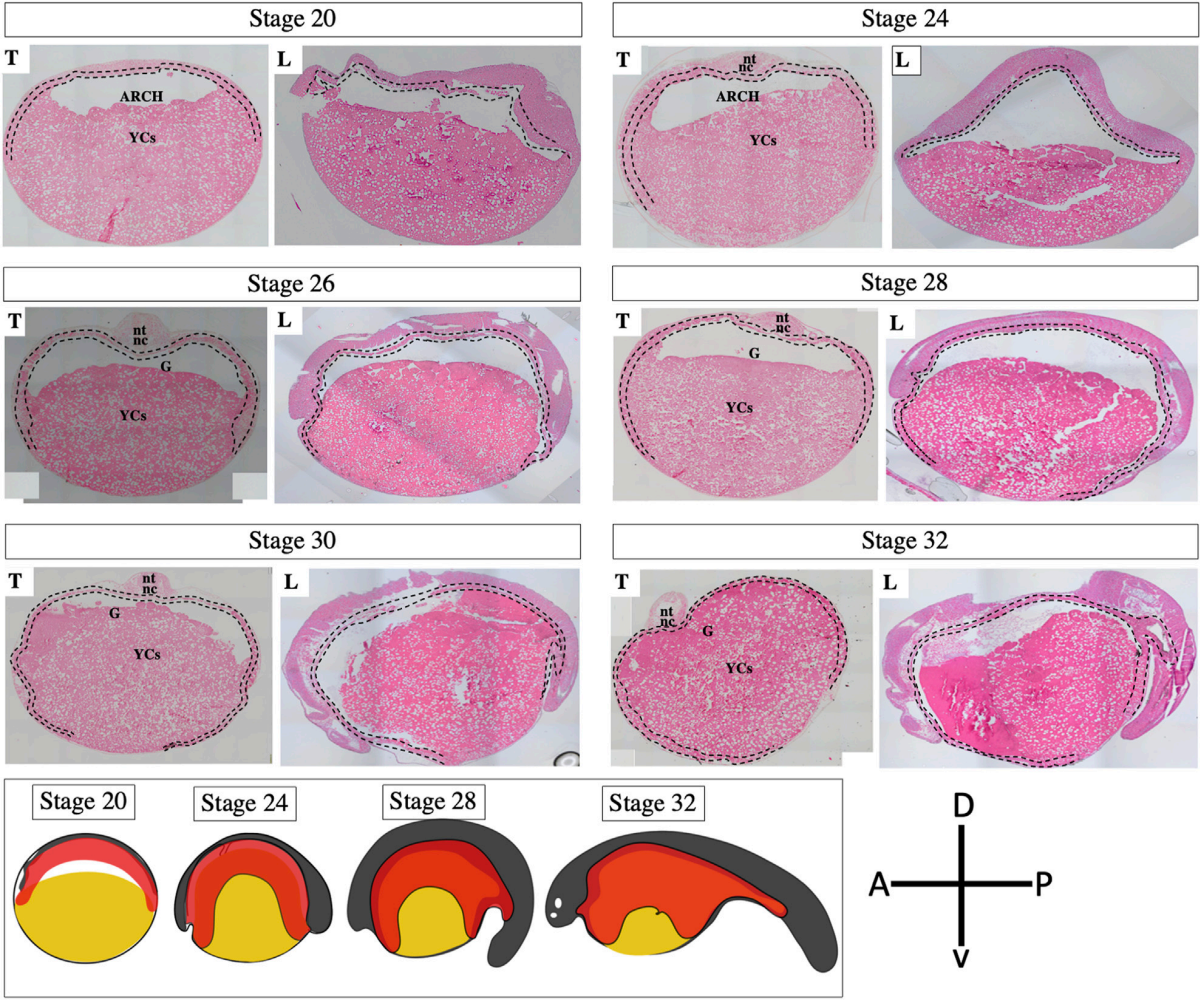
Development of the gut tube in an embryo of a sterlet sturgeon (*Acipenser ruthenus*). Histological sections of sturgeon embryos from stages 20–32 show the development of the gut. During neurulation, the neural field is marginally attached to the roof of the archenteron. The archenteron’s cavity wraps around this protruding floor like an inverted cup, anteriorly, posteriorly, and on both sides (stages 20–24). During pharyngula, the archenteron continues to encompass the entire yolk mass (stages 26–32). Before hatching, whole YCs were found inside the newly developed gut (yolk inside the gut). The scheme below has been drawn on the basis of present results; see Figures 2–5 (Korzhuev and Sharkova, 1967; Ballard and Ginsburg, 1980) and show the encompassment of yolk by the developing gut tube. For magnified picture of these histological observations, please see the Supplementary Figure S4. Yellow color: yolk; red color: gut-endoderm; nt: neural tube; nc: notochord; G: gut; YCs: yolk cells; T: transverse/anterodorsal section; L: lateral cross-section from the head to tail region; dotted line: endodermal cells; A: anterior; P: posterior; D: dorsal; and V: ventral.

The results of our ISH and IHC staining and fate-mapping experiment (which involved CDCFDA labeling via injection and immersion) were consistent with our histological observations (stage 20–32, Figure 6, Supplementary Figures S2, S3), indicating that the encompassment of YCs occurred during the pharyngula stage of embryonic development. Our findings clearly show that sturgeon embryos did not develop a tubular gut on top of a YC mass, nor do ventral YCs of the archenteron contribute to the gut. Instead, sturgeons encompassed their YCs by the developing gut (see Figures 2–6, Supplementary Figure S4).

### 3.2 Post-hatch morphology of the sturgeon gastrointestinal tract and yolk cells

**FIGURE 7:**
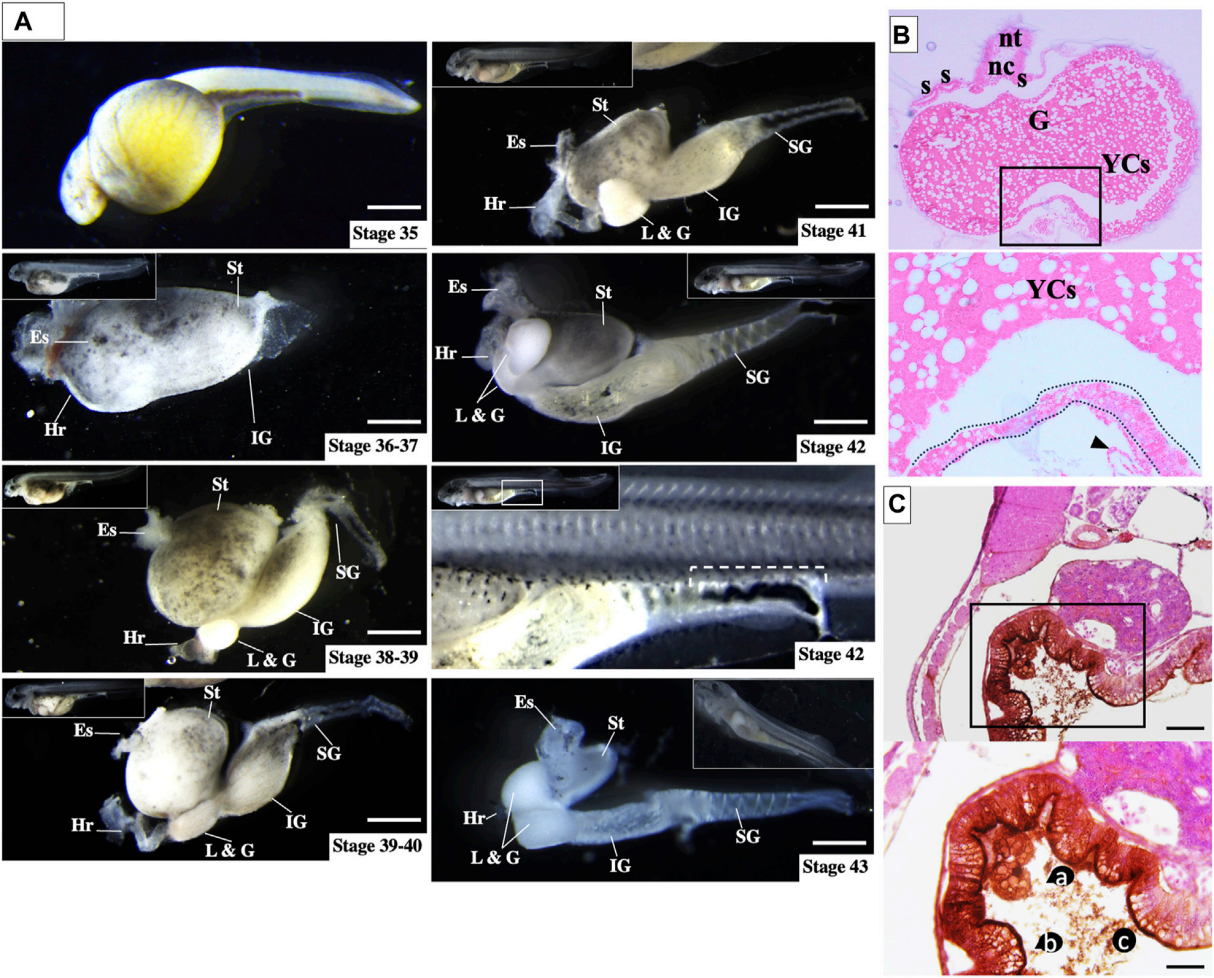
Ontogeny of gut development in sturgeon hatchlings prior to feeding. **(A)** During all stages (stages 36–43), the yolk cells were found inside the gut. However, after stage 36, the yolk cells were found in a broken state inside the gut, and till stage 43, the larvae completed the endogenous nutrition. Stage 42 (rectangular box) shows the rectum with excreta, which indicates that the yolk cells are digested intraintestinally. **(B)** Transverse section of hatched larvae (stage 36) shows the obvious structure of the gut, which encompasses the whole yolk (yolk-inside-gut). **(C)** Immunolabeling of FITC-dextran-labeled part of the gut {(the vegetal blastomere produces the extraembryonic yolk cells; for detail, see Shah et al., 2022}. Immunolabeling of FITC dextran at stage 38 shows YCs were FITC-enriched and broken inside the gut. Es: esophagus; Hr: heart; L & G: liver and gall bladder; IG: intermediate gut; SG: segmented gut; and St: stomach. Scale bars = 1 mm. Inset rectangular box and dotted lines: gut; nt: neural tube; nc: notochord; G: gut; YCs. a: yolk cells, b: enriched-FITC in the inner lining of gut; and c: broken state of YCs. Scale bars indicate 20 μm.

The comparative approach highlights that only sturgeon gut contains a massive amount of yolk inside before hatching (stage 36, Figures 7A and B and Supplementary Figures S2–4, as discussed below). This yolk-inside-gut pattern was accompanied by significant growth of endodermal cells, which encircled a vast area made up of YCs, equivalent to about 3 mm of the abdominal cavity (Figures 3–7). Furthermore, torsion of the gastrointestinal tract was observed immediately after hatching. The abdominal cavity lay anterodorsally to the body, constituting a considerable mass that was loose or excess, with a massive number of YCs (stage 38–42, Figure 7A).

To determine the morphology of YCs inside the gut, we used immunohistochemistry detection of FITC-dextran (vegetal blastomeres/YCs labeled at stage 12) after hatching (stage 38). Almost all FITC-labeled YCs were found to be broken inside the gut, and FITC was enriched on the inner surface of the gut (Figures 7B and C). The broken state of the YCs might be due to enzymatic digestion, as Korzhuev and Sharkova (1967) found enzymatic activity in lecithotrophic stages (before first feeding) of sturgeon (Korzhuev and Sharkova, 1967). This finding supports our observation that broken YCs are utilized for endogenous nutrition (Figure 7C, ac). The presence of excreta in the rectum indicated that the YCs had already been digested and the waste material was being excreted (rectangular box at Stage 42, Figure 7). Moreover, histological analysis showed that by stages 38–40, the stomach and intermediate gut have enclosed the loose and broken YCs by lining the germ layers (Supplementary Figures S2–S4).

## 4 Discussion

### 4.1 Comparison of sturgeon with other taxa

The embryogenesis of sturgeon shares many developmental similarities with *Xenopus* (lobe-finned) rather than with zebrafish (a ray-finned teleost fish), such as holoblastic cleavage leading to cellularization of yolk. During blastulation, the blastocoel separates the ectoderm from the endoderm and permits cell migration. During gastrulation, morphologically distinct “bottle cells” initiate cell involution via the dorsal lip of the blastopore, which leads to the formation of the archenteron (Bolker, 1993; 1994; Collazo et al., 1994). Despite sharing some features with amphibians, sturgeons also have unique characteristics that set them apart from both teleosts and amphibians (Miyake et al., 2006; Saito et al., 2014; Minarik et al., 2017). For example, one of the unique features is their development of primordial germ cells (PGCs). The localization of the germplasm in sturgeon embryos (e.g., indicated by marker genes *dnd*, *vas*, *grip2*, *dzl*, and *nanos*) occurs in the vegetal region similar to that in *Xenopus* (amphibian); however, sturgeon PGC migration patterns are conserved with those of teleosts (Saito et al., 2014; Pocherniaieva et al., 2018; Naraine et al., 2022). Thus, sturgeon shows an intermediate state between those of teleosts and *Xenopus* embryos in terms of PGC localization and migration. In this study, we conducted an analysis of sturgeon gut development, emphasizing its significance within the comparative framework of vertebrates.

### 4.2 Comparison within the ray-finned lineage: sturgeon vs. bichir, gar, and zebrafish

Bichir, sturgeon, gar, and zebrafish belong to the ray-finned fishes (Figure 1). Unlike gar and zebrafish, bichir and sturgeon have holoblastic egg cleavage patterns, which means that they develop the blastocoel and archenteron during blastulation and gastrulation, respectively (Bolker, 1993; 1994; Collazo et al., 1994; Takeuchi et al., 2009b; 2009a). However, a comparative study of gut development between these species has not been conducted yet.

Previously, it was reported that in bichir, the expression of the endoderm marker *sox17* was found in the cells at the dorsal aspect of the archenteron at the late gastrula and neurula stages, but not in YCs (Takeuchi et al., 2009b). Furthermore, *sox17* expression has recently been used to confirm that endodermal cells form a part of the facial structures, known as the pre-oral gut, in bichir, gar, and sturgeon (Minarik et al., 2017). Here, we also did not observe endodermal marker expression in YCs. The expression of Sox17 was present exclusively in endodermal cells up to the late neurula stage (Figure 2). Furthermore, we utilized fibronectin antibody staining, which revealed that the endodermal cells formed the gut, encircling the entire yolk cell mass during development (Figure 3).

**FIGURE 8:**
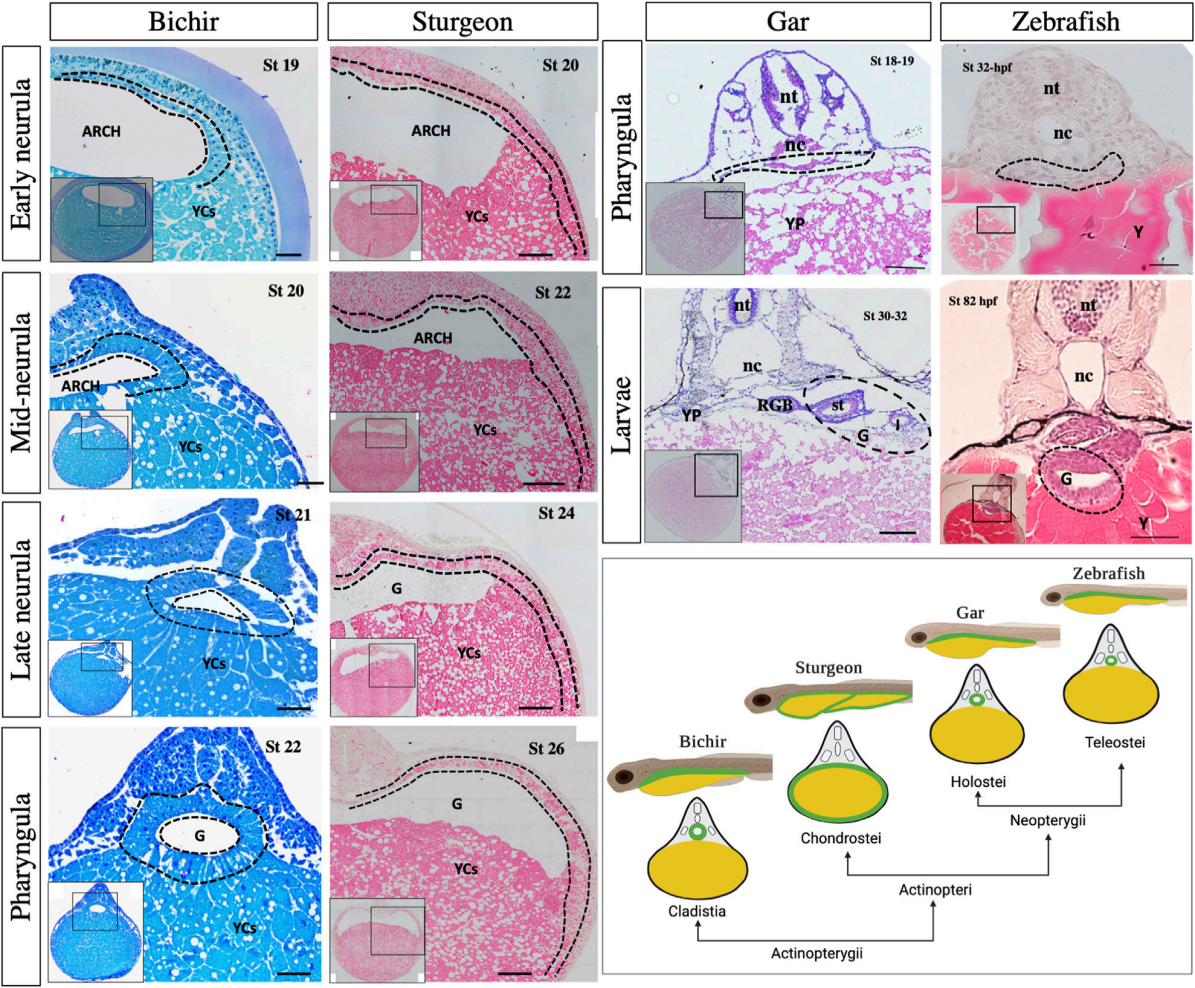
Morphological observation of gut development among bichir, sturgeon, gar, and zebrafish. Bichir: transverse sections of embryo from early to late neurulation (stages 19–22) and pharyngula (stage 26) show the developmental pattern of the gut. During neurulation, the archenteron is seen on the roof of yolk cells. The cells of the archenteron do not move ventrally to encompass the yolk cells. During pharyngula, endoderm cells show the prominent tubular structure of the gut, whereas the yolk is stored in YCs “cellularized form” inside the cellular yolk sac. Sturgeon: transverse sections of embryo from early to late neurulation (stages 20–24) and pharyngula (stage 26) show the early development pattern of the gut. During neurulation, the endodermal cells of the archenteron encompass the semicircle area of yolk mass/cells. During pharyngula, endodermal cells continue to divide and encompass the whole mound of yolk cells. Gar and zebrafish: Transverse sections of gar specimens from pharyngula (stages 18–19) and larvae (stages 30–32) show the morphology of the gut. Stages 18–19 show the endodermal cells on the dorsal position of yolk, which is quite similar to the pharyngula of zebrafish (stage 32 hpf). Similarly, the larval stage of gar (stages 30–32) clearly shows that the gut is a tubular structure on top of huge yolk mass, as seen in zebrafish (stage 82 hpf). There is only a difference in the yolk structure; yolk platelets in gar, and platelet-less yolk in zebrafish. The cartoon image in the right corner depicts gut morphology in larval and cross-sections. Arch: archenteron, nt: neural tube, nt: notochord, G: gut, YCs: yolk cells, dotted lines: endodermal cells, st: stomach, I: intestine, RGB: respiratory gas bladder, YP: yolk platelets, and Y: yolk. Scale bars indicate the 20 μm.

Moreover, our histological analysis of bichir and sturgeon embryos clearly showed the very similar structures of their archenteron during the early neurula stage. Additionally, the ventral part of the archenteron in both species is composed of a massive amount of YCs instead of endodermal cells (Figure 8). These YCs do not contribute to the gut and only provide nutrition as the larvae develop, as also seen in the frog *E. coqui* and in lamprey (Buchholz et al., 2007; Takeuchi et al., 2009b; Shah et al., 2022). During the mid-neurula stage, bichir embryos show that a horizontal archenteron bulge was formed on both sides: internally and rostrolaterally, and this bulge triggers the development of hyoid external gills (Stundl et al., 2019). During late bichir neurulation and pharyngula, the endodermal cells start to develop into the gut cavity on the dorsal position of YCs (bichir stage 21–22, Figure 8). The newly developed gut tube occurs on the roof of a massive amount of YCs. In contrast, the sturgeon’s endodermal cells encompass the YCs, as mentioned above (Figures 1–8 and Supplementary Figures S2–5). According to Takeuchi et al. (2009) and our own findings, bichir and sturgeon embryos with such extracellular vegetal YCs are an evolutionary pre-pattern of gut development, i.e., a YC mass exists on the ventral side of the embryo of bichir and inside the gut of sturgeon (Wallace and Pack, 2003; Takeuchi et al., 2009b). In addition, our pulse-chase experiments with CDCFDA on embryos revealed that after the mid-blastula stage, the YCs do not divide, remain inactive, and are surrounded by gut–endoderm layers. This indicates that YCs are used intraintestinally for nutritional purposes, which is consistent with our previous findings (Shah et al., 2022) (Figure 5, Supplementary Figures S2–5).

Gars cover another important phylogenetic lineage within ray-finned fishes (see Figure 1), but their early gut development remains unknown. Our histological analyses at the pharyngula and larval stages of gar (stages 18–32) clearly showed that the development of the gar gut occurs on the dorsal position of yolk mass, which is, therefore, a tubular structure (Wallace and Pack, 2003; Ng et al., 2005; Takeuchi et al., 2009b; Comabella et al., 2013) (Figure 8). Furthermore, lineage tracing of endodermal cells has revealed that the observed cells were endodermal and formed the tubular gut (Figure 8, Supplementary Figure S6).

Compared to sturgeon and bichir, gar and zebrafish eggs (Neopterygians) contain an uncleaved vegetal pole (Figure 1). In gar and zebrafish, only the animal pole of the egg contributes to develop the gut that sits on the top of the yolk, i.e., a yolk-outside-gut situation. The entire yolk is surrounded by the yolk sac (D’Amico and Cooper, 2001; Comabella et al., 2013) (Figure 8; Supplementary Figure S5). The development of the gut in gar and zebrafish was very similar, with a difference in the structure of the yolk. Gar contains yolk platelets, whereas zebrafish has amorphous yolk. The yolk platelets in gar may be due to fusion of vegetal blastomeres (Figure 8; Supplementary Figure S5) (Shah et al., 2022). In gar and zebrafish, it seems reasonable that the “yolk is outside the gut” because otherwise, the endodermal tissue would be slackened and wrinkled after absorption of the yolk or it would need to be reconstructed drastically to fit the appropriate size as the yolk becomes smaller, as seen in sturgeon (Figure 7). In fact, in many teleosts, the yolk is surrounded by the yolk sac and absorbed via the yolk syncytial layer/vitelline syncytium, and the gut keeps its size compact during the whole process of embryonic development (Long and Ballard, 2001; Wallace and Pack, 2003; Carvalho and Heisenberg, 2010; Comabella et al., 2013).

Compared to all other ray-finned fish lineages, sturgeons develop their gut around the yolk—YCs (as described above)—and utilize it inside the gut. Among ray-finned fishes, this kind of developmental pattern has only been observed in sturgeon so far. Conclusively, the lining of the presumptive sturgeon’s gut and the vitelline syncytium of other fishes have a similar function, i.e., utilization of yolk; however, the mechanism is different, i.e., during the lecithotrophic state, sturgeon utilize yolk materials (YCs) inside the developing gut by using a newly developed digestion system (Korzhuev and Sharkova, 1967). This developmental pattern may be better suited to their environmental conditions or lifestyle and could offer advantages such as enhanced nutrient absorption. Determining why sturgeons have adopted this unique strategy involves complex evolutionary trade-offs and ecological considerations particular to the species. To comprehend the specific evolutionary benefits, one would need to examine the ecological and biological context of sturgeons, including their habitat, feeding patterns, and the evolutionary pressures they have encountered that might favor internal yolk processing.

An additional consideration for this unique gut development may be related to the primordial germ cells (PGCs). PGCs originate elsewhere in the embryo and migrate into the developing gonadal ridges during embryonic development, where they give rise to gametes, eggs and sperm. Our previous research (Saito et al., 2014) indicates that sturgeon PGC specification is akin to that in anurans, whereas their migration pattern is similar to that of teleosts. Given that PGCs in sturgeons are formed at the vegetal pole of the eggs and subsequently have to navigate out of the gut’s encroachment and toward the germinal ridge (Saito et al., 2014), it remains unclear how PGCs are determined alongside the extraembryonic YCs during early development and how they manage to migrate or escape from the gut area designated for digestion. Addressing this query necessitates a detailed comparative study of PGCs and gut development between teleost and non-teleost species, particularly since the PGC development in gars and bichirs remains to be elucidated.

### 4.3 Comparison to the lobe-finned lineage: sturgeon vs. *Xenopus*, chicken, and mice

**FIGURE 9:**
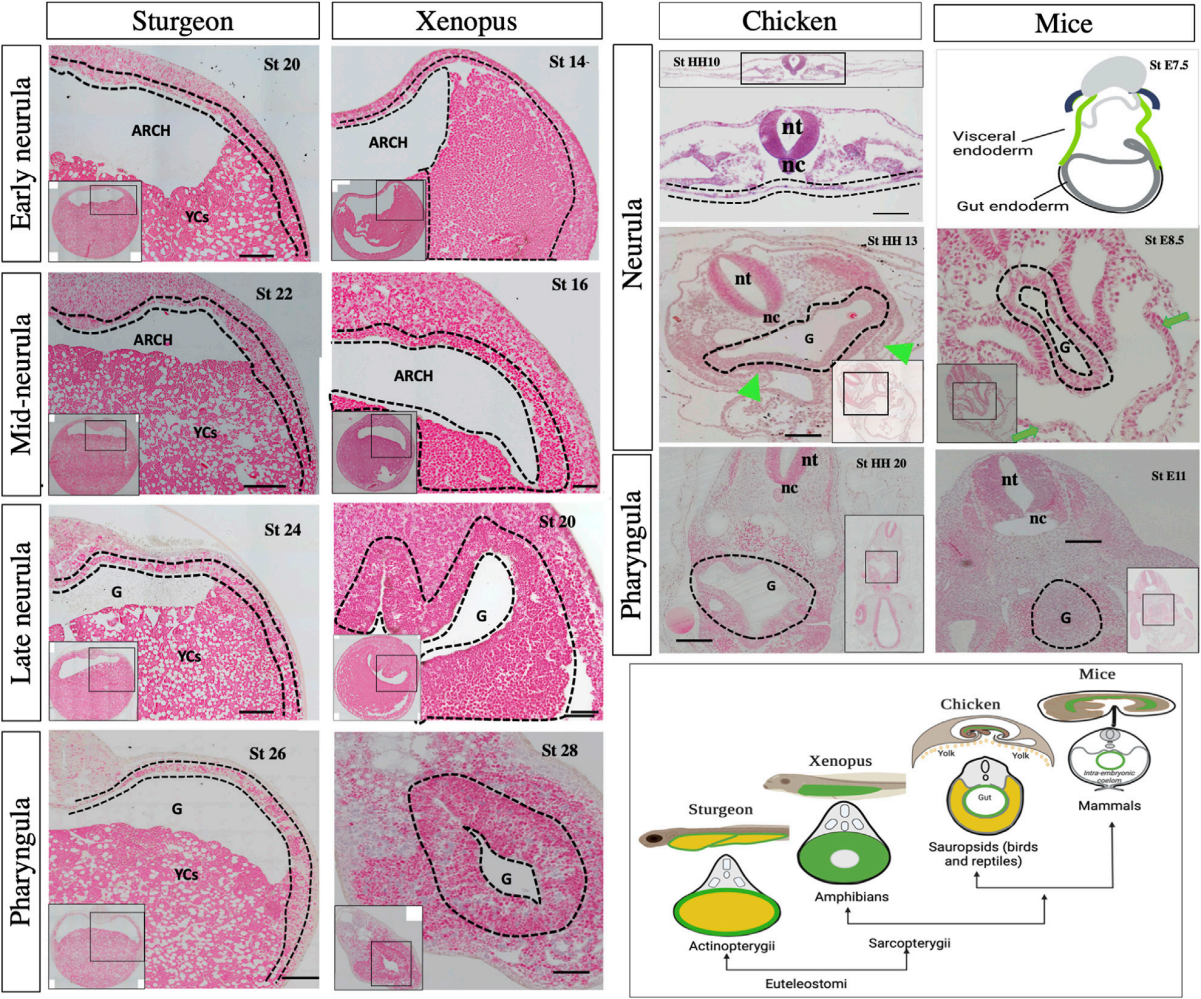
Morphological observation of gut development among sturgeon, *Xenopus*, chicken, and mice. Sturgeon: the pattern of early gut development in sturgeon (see legend of Figure 8). *Xenopus*: transverse sections of the embryo from early to late neurulation (stages 13–20) and pharyngula (stage 28) shows the development of the gut. During the neurulation, the archenteron shows the same structure as in sturgeon; however, the cells from the ventral side of the archenteron are endoderm (yolk endoderm cells). During pharyngula, the gut shows the prominent tubular structure of gut-endoderm, whereas yolk is intracellular (inside the endodermal cells). Chicken: transverse sections of embryos from neurula (stage HH10–HH13) to pharyngula (stage HH20) show the development of the gut. During neurula, a flattened endoderm layer is localized above the yolk mass and below the mesoderm/nerve cord. Embryos also show the obvious structure of the tubular gut and extraembryonic layer (Gilbert, 2010; Frankenberg, 2012). Mice: drawing of very early neurula (stage 7.5) and transverse sections of mid-neurula (stage 8.5) and pharyngula (stage 11.5) shows the development of the gut. During the neurula stage, visceral endoderm (green color) developed the extraembryonic layers, indicated by green arrows. Gray color shows the definitive endoderm, which forms the gut tube (Frankenberg, 2012; Nowotschin et al., 2019a; Nowotschin et al., 2019b). The cartoon image in the right corner depicts gut morphology in larval and cross-sections. Arch: archenteron, nt: neural tube, nc: notochord, G: gut, YCs: yolk cells, dotted lines: endodermal cells, and green arrows: extraembryonic layers. Scale bars indicate the 20 μm.

Additionally, it is also necessary to compare the sturgeon with lobe-finned representatives, including *Xenopus*, chicken, and mice, as sturgeon shares many developmental similarities with *Xenopus* (Bolker, 1993; 1994; Collazo et al., 1994). The histological analysis from the neurula to pharyngula stages between sturgeon and *Xenopus* showed obvious differences in the case of gut development (Figure 9). For example, *Xenopus* and sturgeon embryos at the neurula stage clearly showed that the endoderm lines the archenteron cavity. Like in sturgeon, the dorsal endoderm of *Xenopus* is made up of a single layer of cells. In contrast, the ventral endoderm of *Xenopus* is made up of several layers of large endodermal cells (with intracellular yolk platelets), while in sturgeons, it is composed of extra-embryonic YCs instead of endodermal cells. In *Xenopus*, from the mid-neurula to the pharyngula stage, the archenteron gradually closes, and the gut cavity is a continuation of the archenteron and laid with mesenchyme. The definitive gut cavity is formed, containing the cells of the original archenteron as well as the more ventral endoderm, and the cells contain yolk in form yolk platelets and utilize them intracellularlly (*Xenopus*, Figure 9). In case of sturgeons, endodermal cells from the archenteron move ventrolaterally to encompass the YCs; however, yolk in the form of YCs is utilized intra-intestinally (Figures 2–9 and Supplementary Figures S2–5).

Compared to that of sturgeons, the chicken (e.g., amniotes) egg has an uncleaved vegetal pole. The entire gut is developed and laid on top of the yolk sac, as seen in zebrafish (Figure 1). However, the extra-embryonic membranes of the chick are four in number: the yolk-sac, the amnion, the serosa, and the allantois. The splanchnopleuric mesenchyme is composed of the mesoderm external to the coelom plus the endoderm (Gilbert, 2010). The splanchnopleure, instead of forming a closed gut, grows over the yolk and becomes a yolk sac (chicken, Figure 9). This yolk region is in contact with the midgut and primitive gut present above the yolk (chicken, Figure 9). Comparatively, in sturgeons, endodermal cells from the archenteron encompass YCs for endogenous nutrition, which appears like the splanchnopleure-like structure of chicken. However, sturgeons do not develop the extra embryonic layers and retain a unique mode of gut development pattern to utilize the yolk (Figures 2–9 and Supplementary Figures S2–5).

In addition to a volume of yolk associated with development of gut endoderm, the embryo of mammals (e.g., mice) has less/no yolk and retains a holoblastic cleavage pattern. In mice, the gut endoderm forms on the surface of the embryo at gastrulation, where definitive endoderm cells (derived from the epiblast) intercalate into the overlying visceral endoderm (derived from the primitive endoderm) and form the extraembryonic tissues (Nowotschin et al., 2019a). The developing gut tube epithelium forms the definitive endoderm along the anterior–posterior axis (middle, E8.5). At this point in time, the anterior endoderm has formed the foregut tube, the posterior endoderm has formed the hindgut tube, and the middle region forms the midgut. Gut tube formation is completed by E9.5 in the mouse embryo {(Frankenberg, 2012; Nowotschin et al., 2019b). mice, Figure 9}. Due to the absence of a yolk and the retention of extraembryonic layers in mammals, the comparison between the embryogenesis and gut development of mice and sturgeon is significantly different.

In conclusion, our study provides new insights into the development of the gut in sturgeons, one of the oldest extant fish groups. Our findings demonstrate that gut development in sturgeons differs from that of the above-mentioned vertebrates, including model and non-model organisms. In most vertebrates, maternally provided yolk is absorbed either intracellularly by the endoderm cells (e.g., *Xenopus*) or extracellularly by the yolk sac and transported to the gut (e.g., bichir, zebrafish, and chicken). In any case, the digestive tract starts its function with digestion of exogenous nutrition after the lecithotrophic state (after first feeding). In contrast, in the unique case of sturgeons, they digest their endogenous nutrition (vegetal cells containing yolk platelets; YCs) inside of their newly developed digestive system and start excretion from their gut already before feeding (Figure 6, stage 42). Thus, it is possible that the sturgeon evolved a unique pattern of gut development compared to the abovementioned other vertebrate lineages (see heading 4.2) (Figures 1–9).

## Data Availability

Publicly available datasets were analyzed in this study. This data can be found here: https://www.ncbi.nlm.nih.gov/gene/117394216 https://www.ncbi.nlm.nih.gov/gene/?term&equals;117431529.
