# Supplementary material for: Sturgeon gut development: a unique yolk utilization strategy among vertebrates
Source: Front Cell Dev Biol. 2024 May 30;12:1358702. doi: 10.3389/fcell.2024.1358702 (PMC11169612; doi:10.3389/fcell.2024.1358702)
Supplement: Supplementary file 1 [file DataSheet2.docx]

Supplementary Material

# Supplementary Figures

| 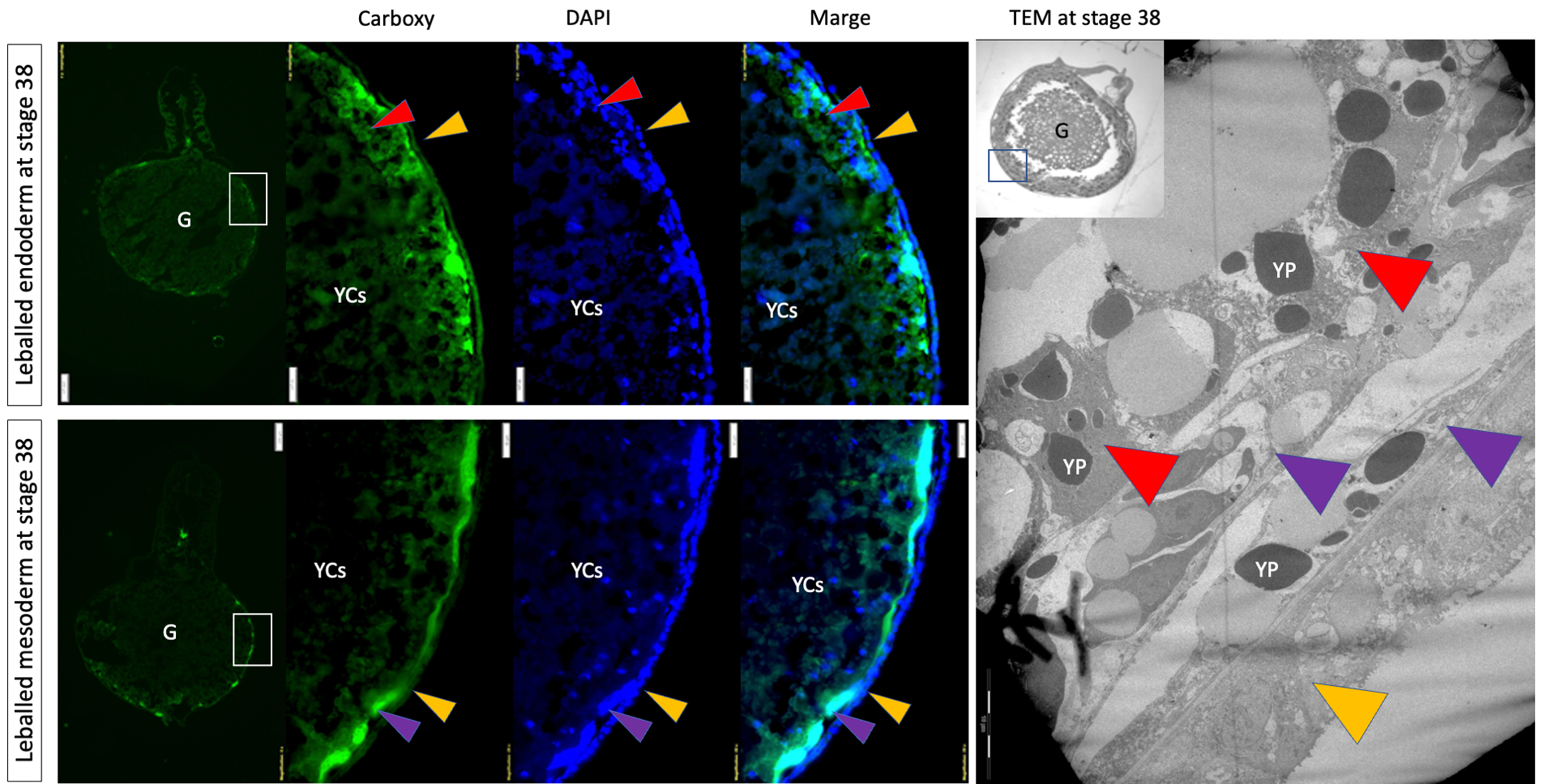 |
| --- |

**Figure S1. Mesoderm and endoderm labelling**

The figure shows the staining of the endodermal and mesodermal layers using carboxy-CDCFDA on the dorsoventral sections of hatched larvae. Although labelling only the endodermal cells without also labelling the mesodermal cells was quite challenging, it clearly aligned with the ultrastructure of the germ layers at the same developmental stages using an electron microscope. Yellow arrow – ectoderm, purple arrow – mesoderm, red arrow – endoderm, YCs – Yolk cells, G – gut.

| 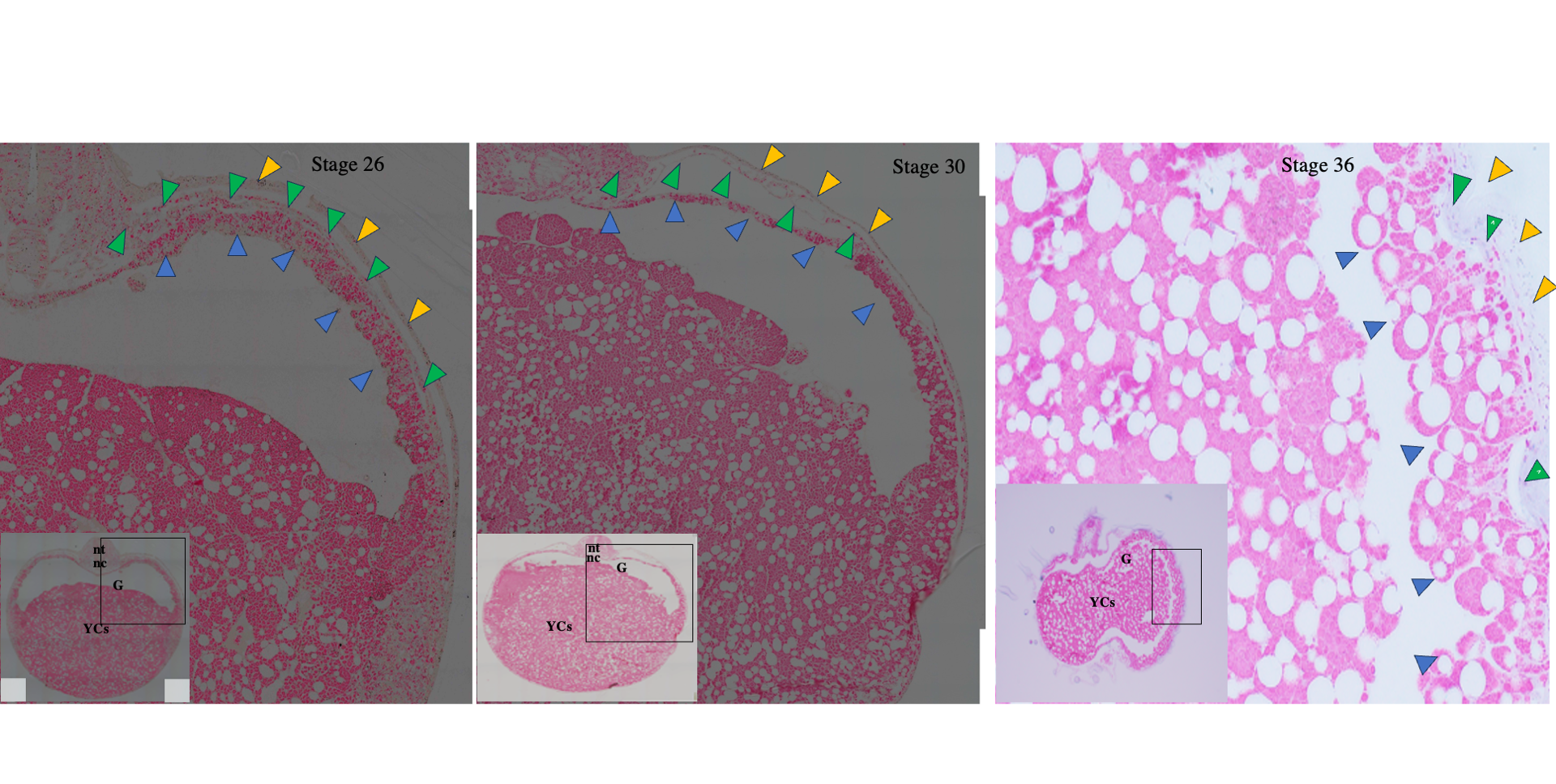 |
| --- |

**Figure S2. Magnified histological pictures of germ layers encompassing the yolk cells.**

The pictures show the obvious structure of three germ layers endoderm, mesoderm, and ectoderm. Blue arrow – endoderm, green arrow – mesoderm, yellow arrow – ectoderm. YCs – Yolk cells, G – gut, nt—neural tube, nc—notochord. Scale bars indicate 10 μm

| 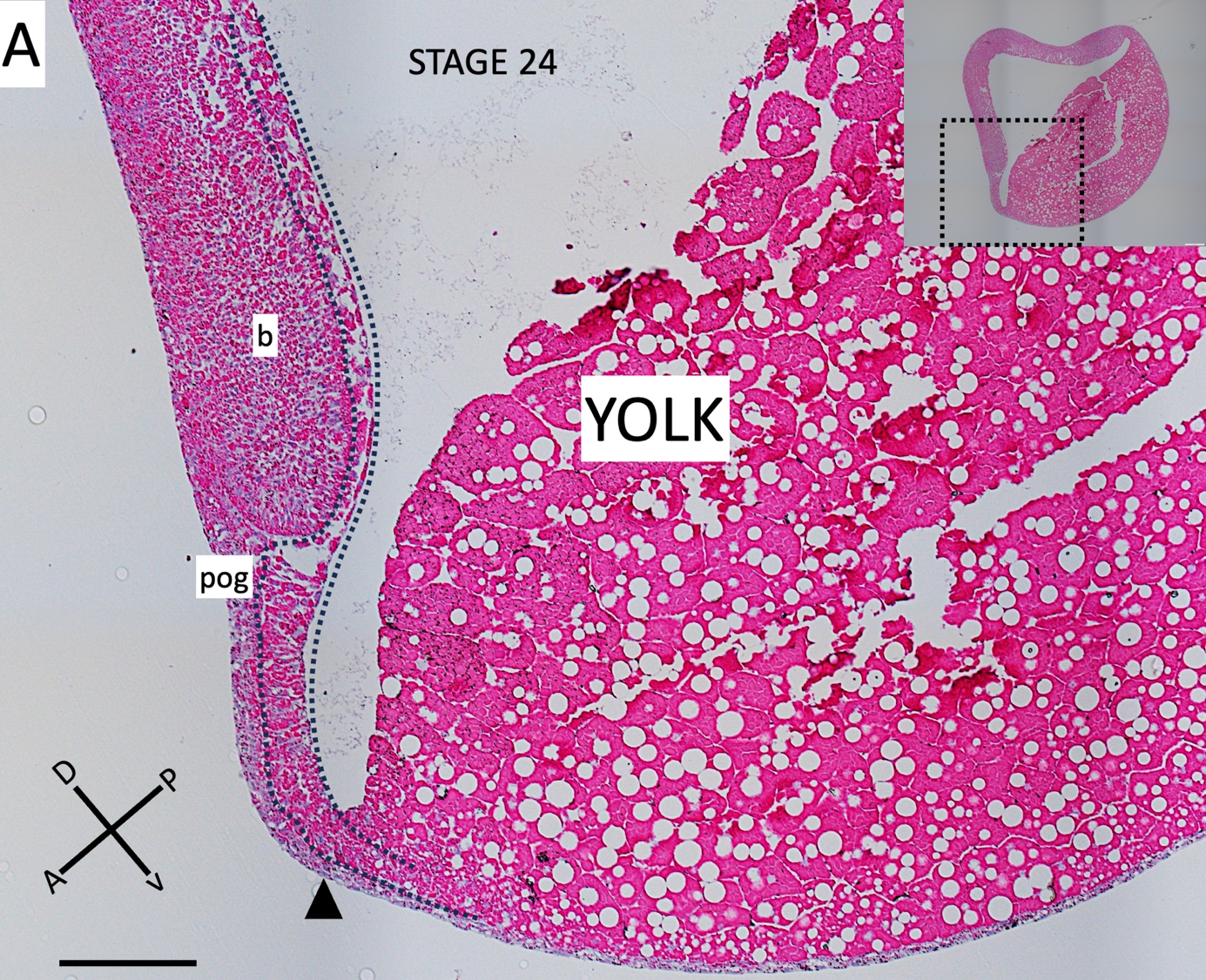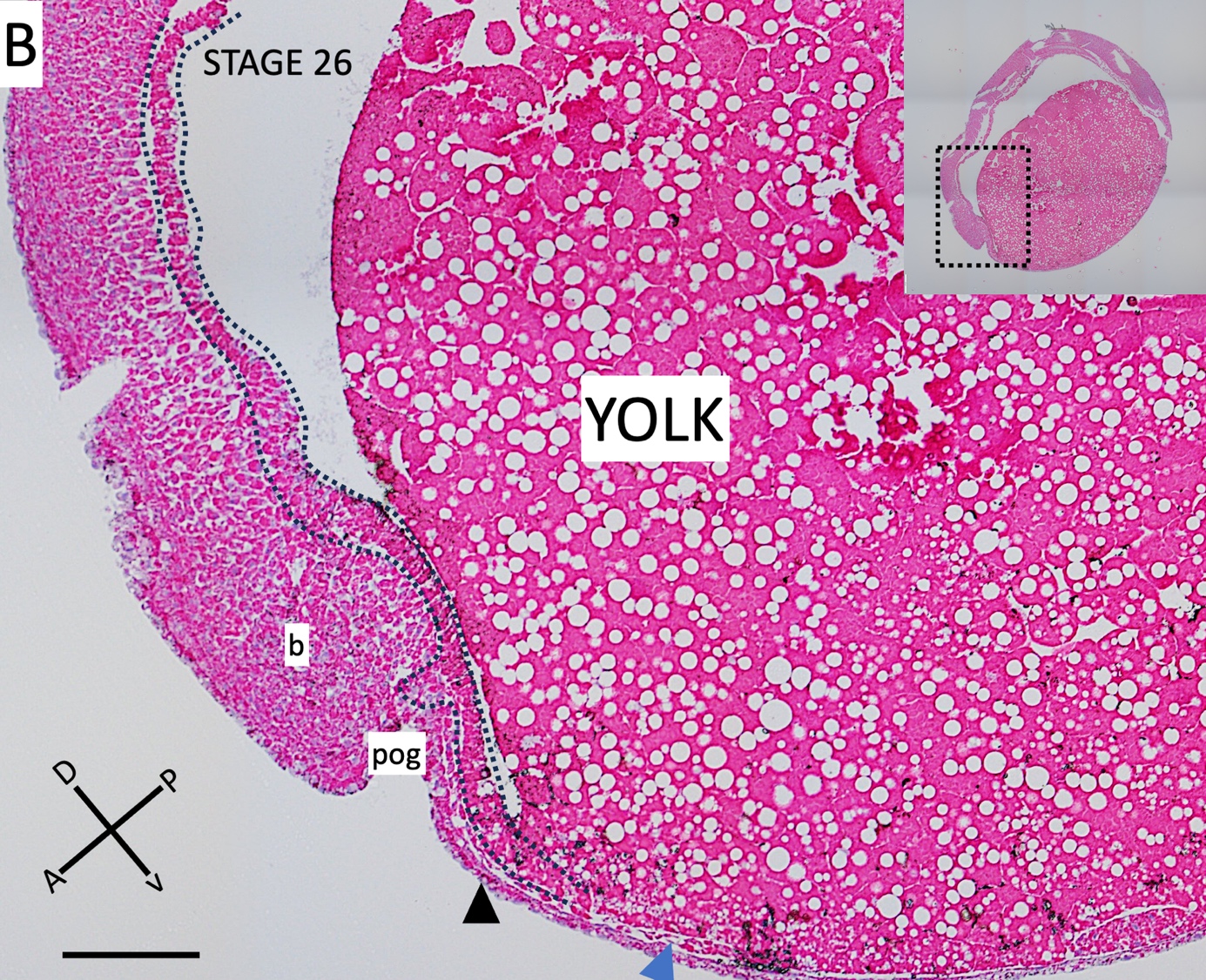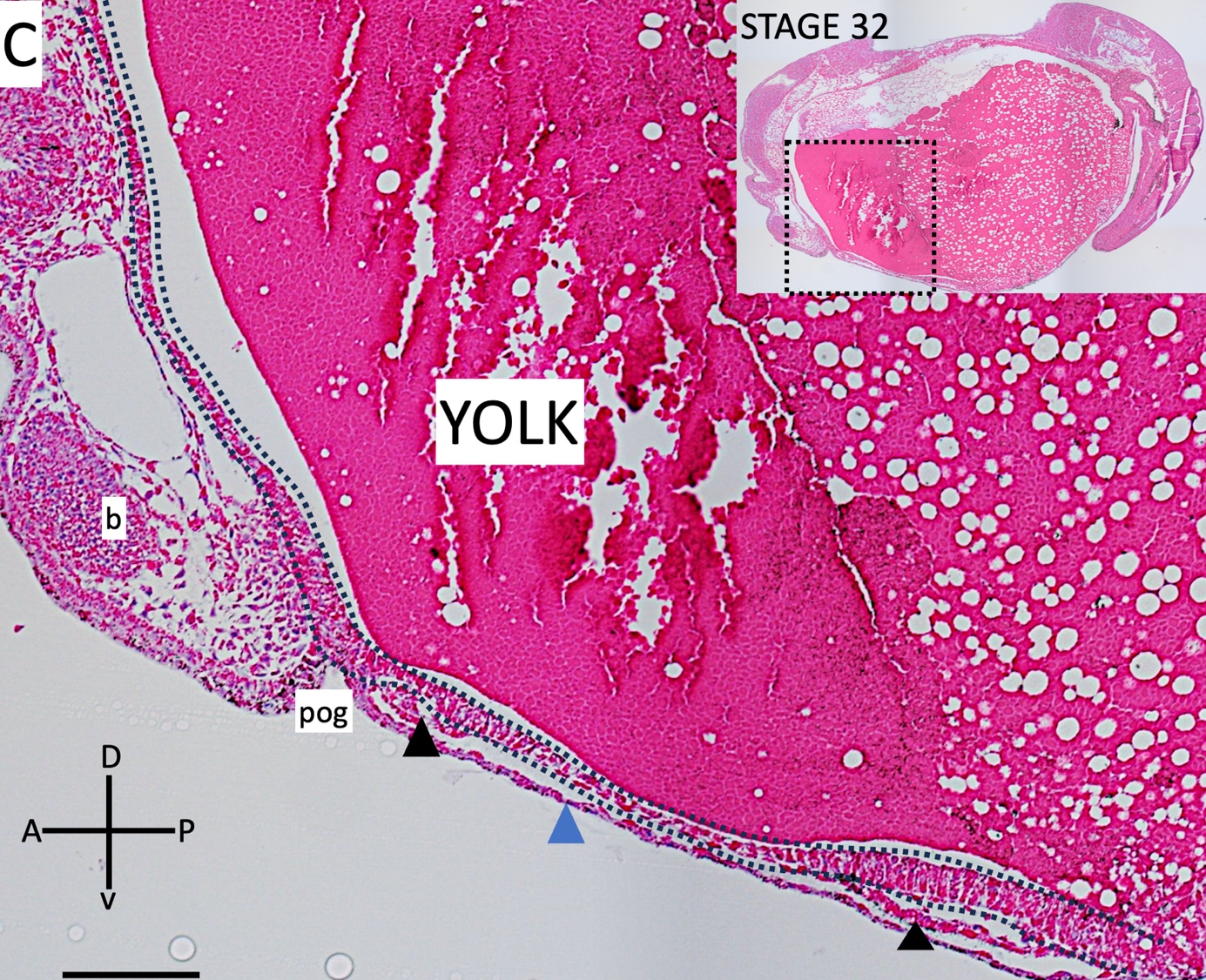  **Figure S3A-C: Enlarged Histological Sections of Embryonic Germ Layers from a Transverse View"**  **Figure S3A-C** presents a transverse section of an embryos at neurula (a) stage 24, pharungula (b) stage 26 and tail but (c) stage 32. These histilogical analysis highlighting cellular and tissue structures. On the right, an overview of the entire embryos in a sagittal section is provided. The magnified section, within a dashed box, reveals a prominent area of YOLK material encircled by distinct tissue types and cellular organization. The endodermal layer surrounding the yolk is demarcated by a double-dotted line. The labels are as follows: 'b' for brain, 'pog' for pre-oral gut. The black arrow points to the mesoderm, and the blue arrow indicates the ectoderm. Orientation markers are given at the bottom: 'A' for anterior, 'P' for posterior, 'D' for dorsal, and 'V' for ventral. Scale bars indicate 200 μm. |
| --- |

| **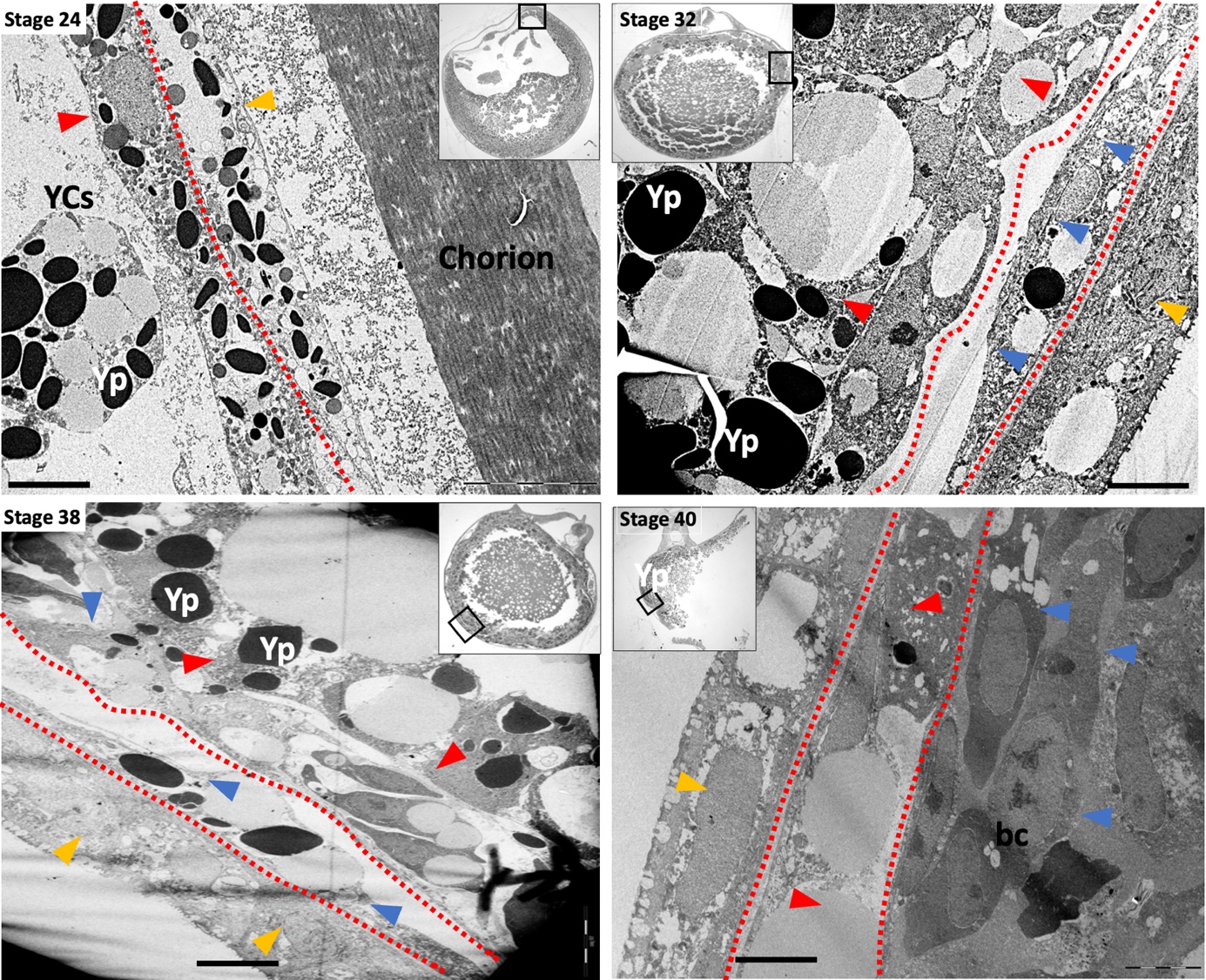**  **Figure S4. Transmission electron microscopy of sturgeon embryo’s germ layers**  Transverse sections of embryos from late neurula (stage 24), tailbud (stage 32), and hatched larvae (stage 38-40) reveal the ultrastructure of the germ layer developmental pattern. Endoderm cells were observed over YCs until neurula. From the tailbud to the hatching larvae, the lateral and ventral sides (rectangular box) show the visible structure of germ layers with broken and loos YCs. The dotted lines separate the germ layers. Yellow arrow / outer layer — ectoderm, blue arrow / middle layer — mesoderm, red arrow / inner layer — endoderm possibility with some secretory cells and enteric nervous system cells, Yp — yolk platelets, YCs — yolk cells, bc — blood cells. Scale bars indicate 10 μm. |
| --- |

| 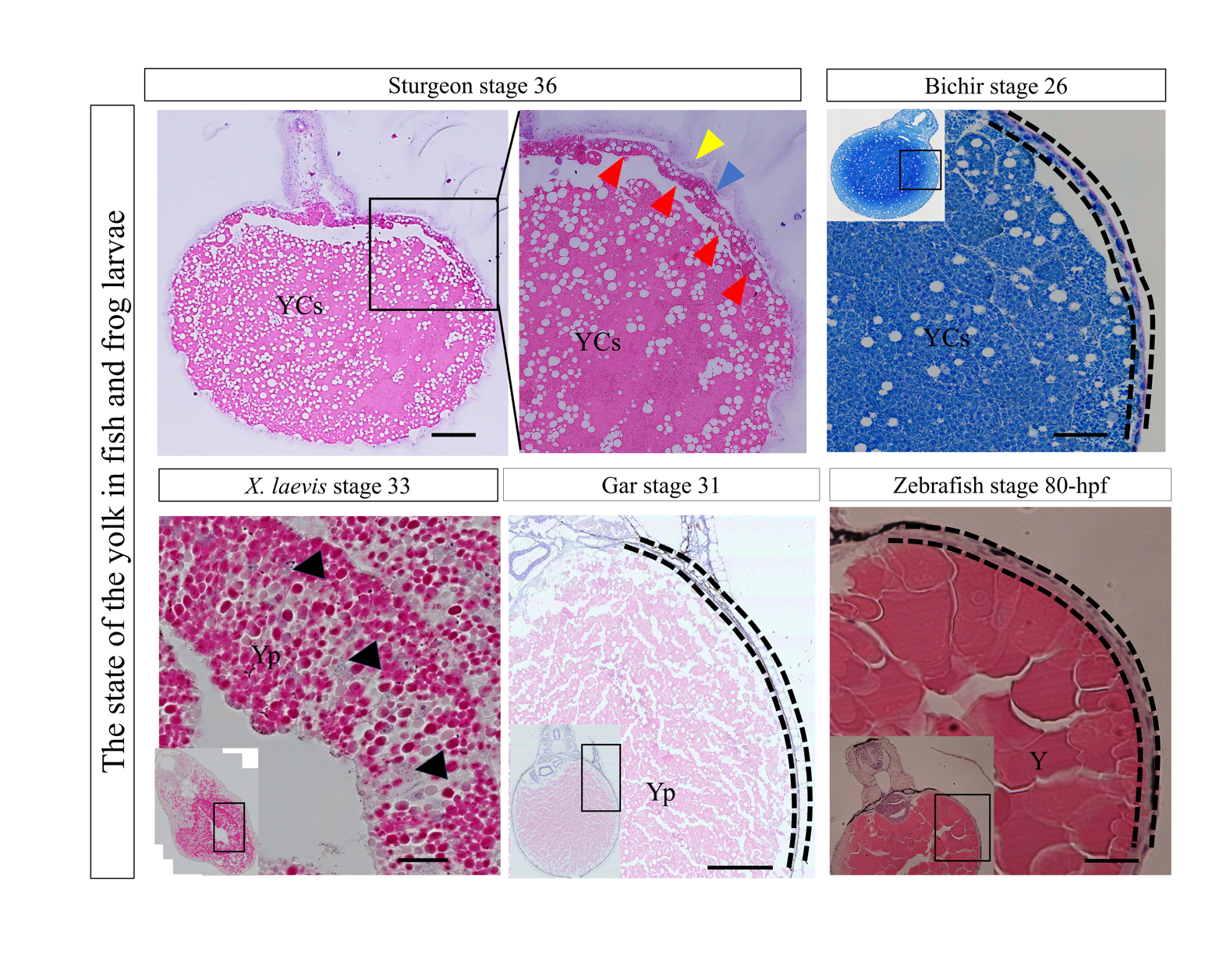 |
| --- |

**Figure S5.** Magnified view of yolk surrounded by gut in the larvae of sturgeon (indicated by rectangular box). Yellow arrow — ectoderm, blue arrow — mesoderm, red arrow — endoderm, black arrows — nuclei in endodermal cells. In comparison, in other fishes including bichir, gar, and zebrafish, the yolk is surrounded by ectoderm (indicated by black dotted line). On the other hand, in *Xenopus*, the yolk is intracellular (indicated by black arrows). YCs – yolk cells, Yp – yolk platelets, Y – yolk. Scale bars indicate 10 μm.

| 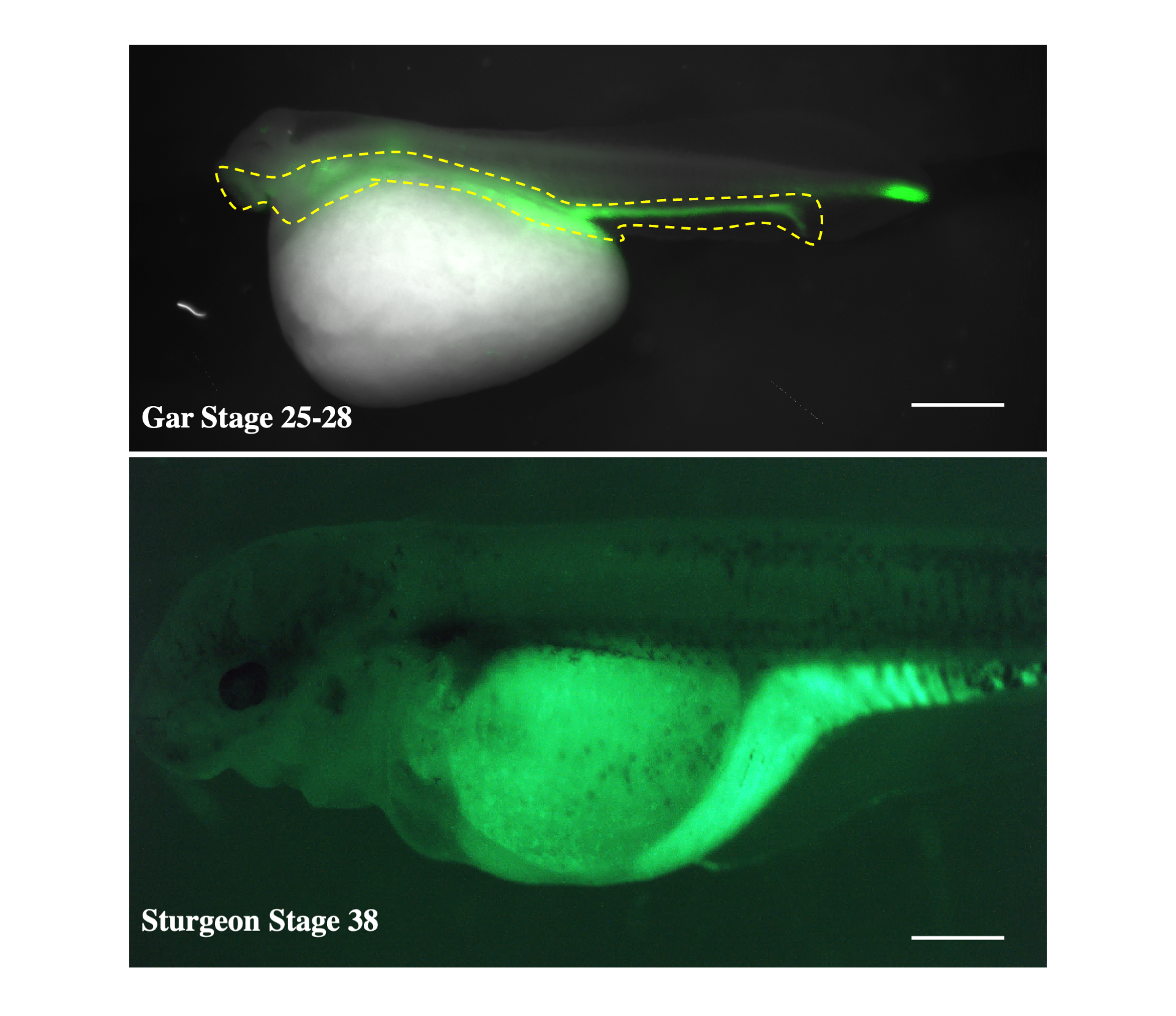 |
| --- |

**Figure S6. In vivo labelling of gut-endoderm of gar and sturgeon**

The endodermal cells of gar and sturgeon were labelled at neurula stage and allowed to develop till hatching. The picture of labelled specimens of was gift from (﻿Department of Zoology, Charles University, Prague, Czech Republic). The pictures ware taken using fluorescence stereomicroscope Olympus. Dotted line indicates the positive labelling of endoderm cells, whereas some ectodermal cells were also labelled during the injection that shows staining on tail region (Minarik et al., 2017). In comparison to sturgeon, gar gut developed on the dorsal position of the yolk, whereas sturgeon gut developed around the yolk cells. Scale bars indicate 2 mm.

**
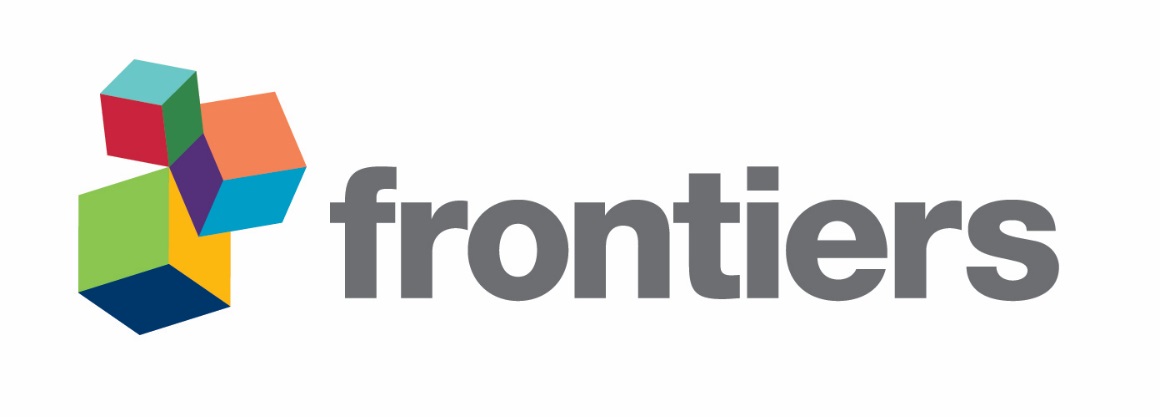
**
