# Supplementary material for: Sturgeon gut development: a unique yolk utilization strategy among vertebrates
Source: Front Cell Dev Biol. 2024 May 30;12:1358702. doi: 10.3389/fcell.2024.1358702 (PMC11169612; doi:10.3389/fcell.2024.1358702)
Supplement: Supplementary file 2 [file DataSheet1.docx]

Supplementary Material

# Supplementary Figures

| 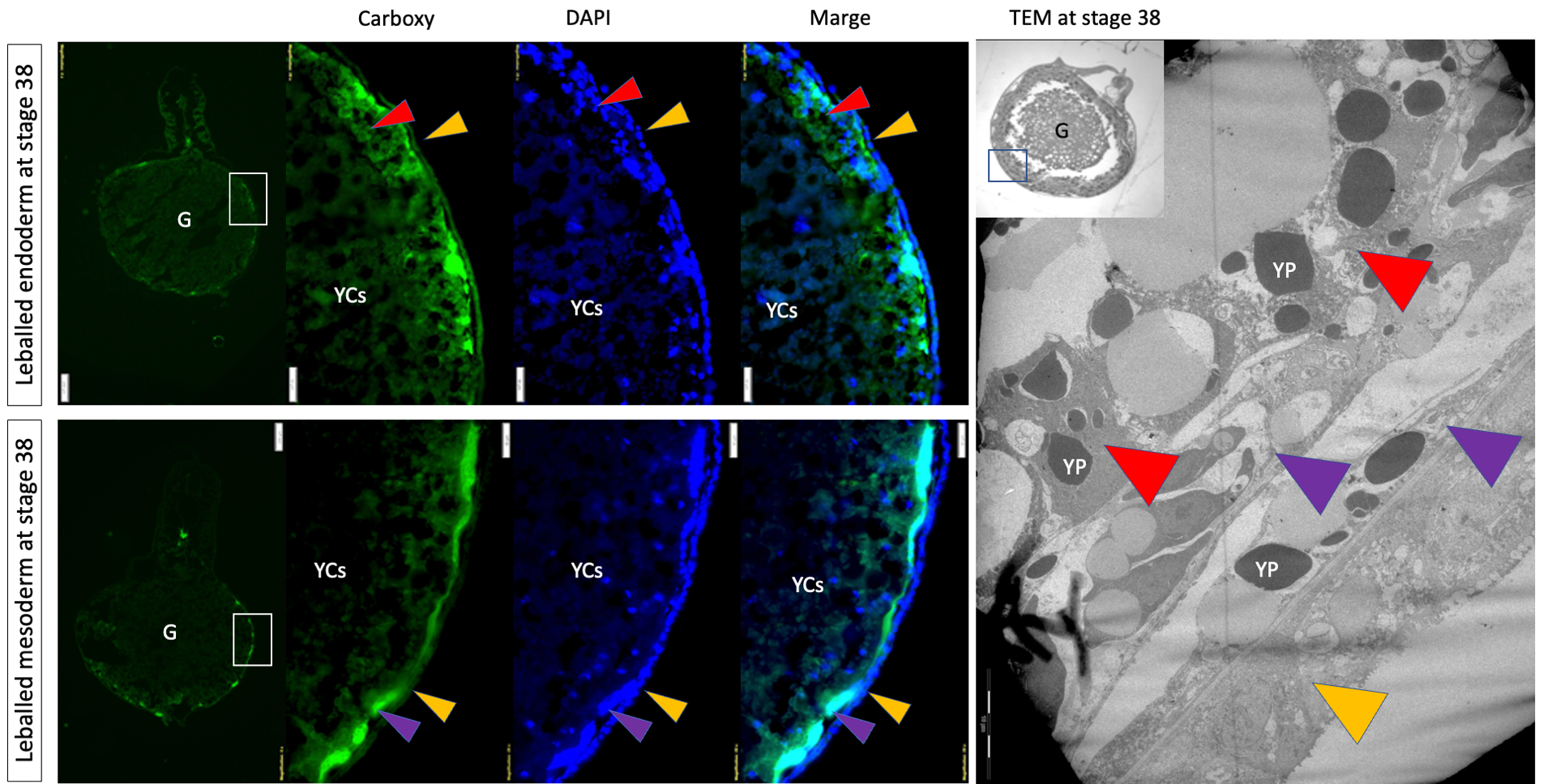 |
| --- |

**Figure S1. Mesoderm and endoderm labelling**

The figure shows the staining of the endodermal and mesodermal layers using carboxy-CDCFDA on the dorsoventral sections of hatched larvae. Although labelling only the endodermal cells without also labelling the mesodermal cells was quite challenging, it clearly aligned with the ultrastructure of the germ layers at the same developmental stages using an electron microscope. Yellow arrow – ectoderm, purple arrow – mesoderm, red arrow – endoderm, YCs – Yolk cells, G – gut.

| 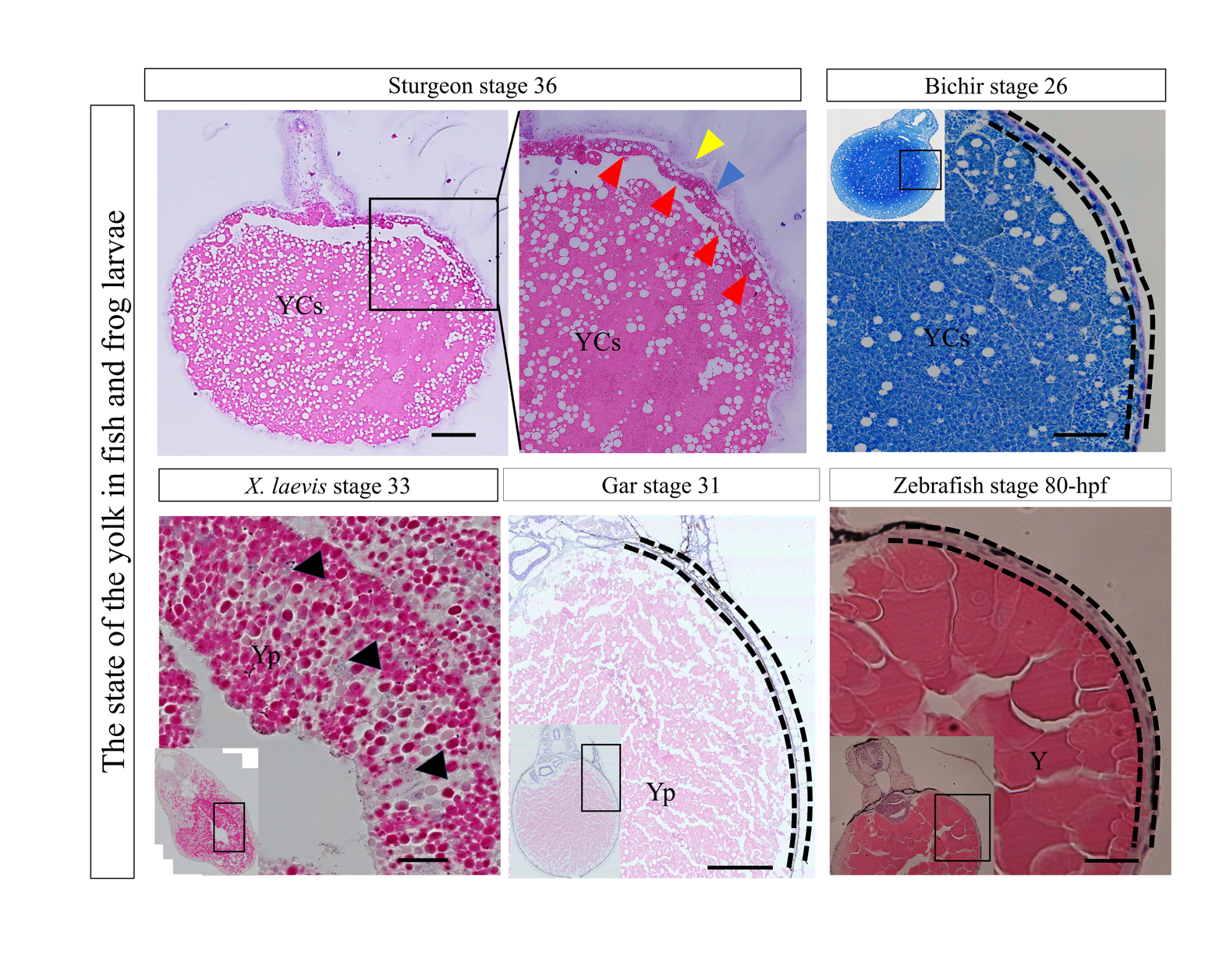 |
| --- |

**Figure S2.** Magnified view of yolk surrounded by gut in the larvae of sturgeon (indicated by rectangular box). Yellow arrow — ectoderm, blue arrow — mesoderm, red arrow — endoderm, black arrows — nuclei in endodermal cells. In comparison, in other fishes including bichir, gar, and zebrafish, the yolk is surrounded by ectoderm (indicated by black dotted line). On the other hand, in *Xenopus*, the yolk is intracellular (indicated by black arrows). YCs – yolk cells, Yp – yolk platelets, Y – yolk.

| 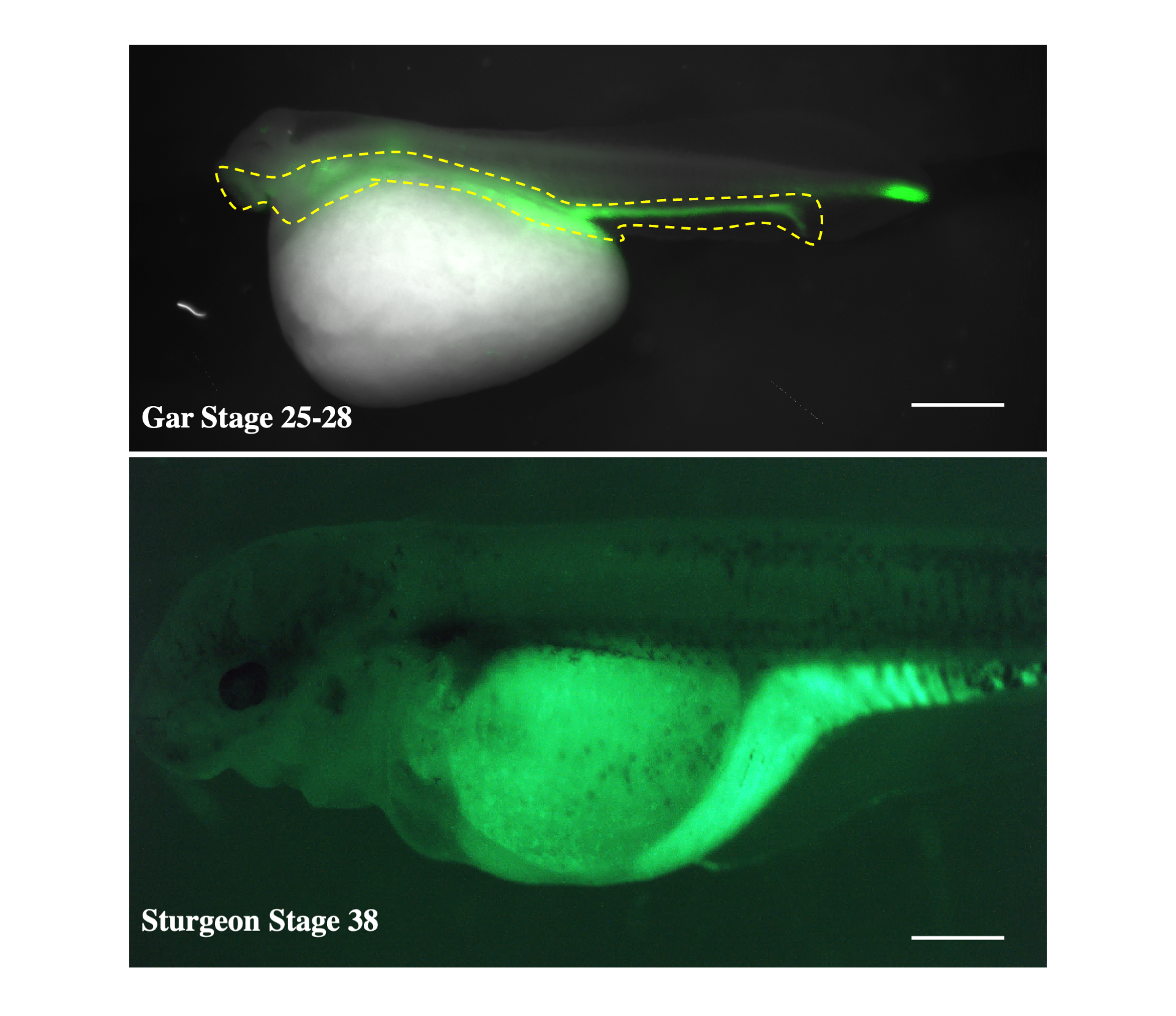 |
| --- |

**Figure S3. In vivo labelling of gut-endoderm of gar and sturgeon**

The endodermal cells of gar and sturgeon were labelled at neurula stage and allowed to develop till hatching. The picture of labelled specimens of was gift from (﻿Department of Zoology, Charles University, Prague, Czech Republic). The pictures ware taken using fluorescence stereomicroscope Olympus. Dotted line indicates the positive labelling of endoderm cells, whereas some ectodermal cells were also labelled during the injection that shows staining on tail region (Minarik et al., 2017). In comparison to sturgeon, gar gut developed on the dorsal position of the yolk, whereas sturgeon gut developed around the yolk cells.

| 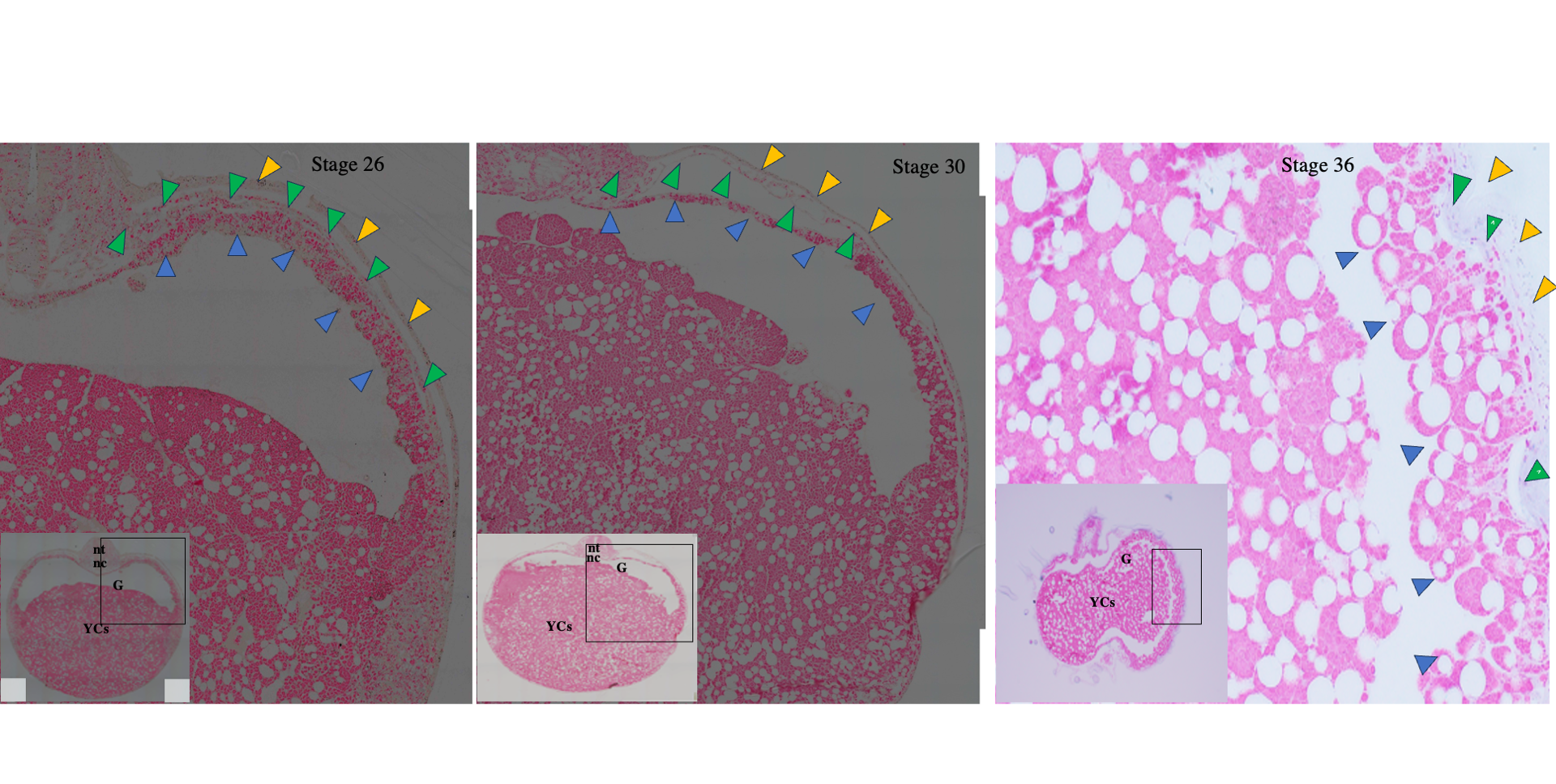 |
| --- |

**Figure S4. Magnified histological pictures of germ layers encompassing the yolk cells.**

The pictures show the obvious structure of three germ layers endoderm, mesoderm, and ectoderm. Blue arrow – endoderm, green arrow – mesoderm, yellow arrow – ectoderm. YCs – Yolk cells, G – gut, nt—neural tube, nc—notochord.

**
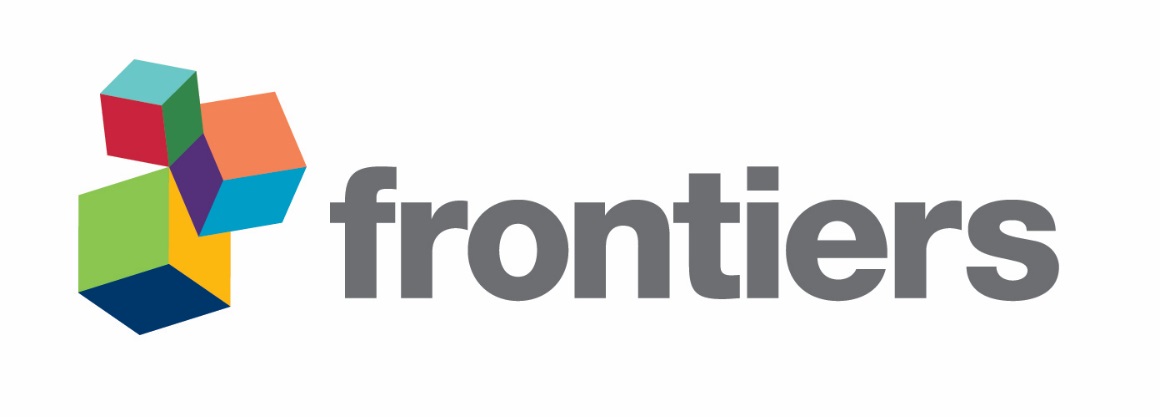
**
